# Long Term Development of Gut Microbiota Composition in Atopic Children: Impact of Probiotics

**DOI:** 10.1371/journal.pone.0137681

**Published:** 2015-09-17

**Authors:** N. B. M. M. Rutten, D. M. W. Gorissen, A. Eck, L. E. M. Niers, A. M. Vlieger, I. Besseling-van der Vaart, A. E. Budding, P. H. M. Savelkoul, C. K. van der Ent, G. T. Rijkers

**Affiliations:** 1 Department of Pediatric Pulmonology and Allergology, Wilhelmina Children’s Hospital, University Medical Centre, Utrecht, the Netherlands; 2 Department of Pediatrics, St Antonius Hospital, Nieuwegein, the Netherlands; 3 Department of Medical Microbiology and Infection Control, VU University Medical Centre, Amsterdam, the Netherlands; 4 Winclove Probiotics BV, Amsterdam, the Netherlands; 5 Department of Medical Microbiology, School of Nutrition & Translational Research in Metabolism (NUTRIM), Maastricht University Medical Centre, Maastricht, the Netherlands; 6 Department of Sciences, University College Roosevelt Academy, Middelburg and Department of Medical Microbiology and Immunology, St Antonius Hospital, Nieuwegein, the Netherlands; GI Lab, UNITED STATES

## Abstract

**Introduction:**

Imbalance of the human gut microbiota in early childhood is suggested as a risk factor for immune-mediated disorders such as allergies. With the objective to modulate the intestinal microbiota, probiotic supplementation during infancy has been used for prevention of allergic diseases in infants, with variable success. However, not much is known about the long-term consequences of neonatal use of probiotics on the microbiota composition. The aim of this study was to assess the composition and microbial diversity in stool samples of infants at high-risk for atopic disease, from birth onwards to six years of age, who were treated with probiotics or placebo during the first year of life.

**Methods:**

In a double-blind, randomized, placebo-controlled trial, a probiotic mixture consisting of *B*. *bifidum* W23, *B*. *lactis* W52 and *Lc*. *Lactis* W58 (Ecologic® Panda) was administered to pregnant women during the last 6 weeks of pregnancy and to their offspring during the first year of life. During follow-up, faecal samples were collected from 99 children over a 6-year period with the following time points: first week, second week, first month, three months, first year, eighteen months, two years and six years. Bacterial profiling was performed by IS-pro. Differences in bacterial abundance and diversity were assessed by conventional statistics.

**Results:**

The presence of the supplemented probiotic strains in faecal samples was confirmed, and the probiotic strains had a higher abundance and prevalence in the probiotic group during supplementation. Only minor and short term differences in composition of microbiota were found between the probiotic and placebo group and between children with or without atopy. The diversity of Bacteroidetes was significantly higher after two weeks in the placebo group, and at the age of two years atopic children had a significantly higher Proteobacteria diversity (p < 0.05). Gut microbiota development continued between two and six years, whereby microbiota composition at phylum level evolved more and more towards an adult-like configuration.

**Conclusion:**

Perinatal supplementation with Ecologic® Panda, to children at high-risk for atopic disease, had minor effects on gut microbiota composition during the supplementation period. No long lasting differences were identified. Regardless of intervention or atopic disease status, children had a shared microbiota development over time determined by age that continued to develop between two and six years.

## Introduction

The gastrointestinal tract is home to a diverse microbiota of about 10^14^ bacteria, representing up to 1500 bacterial species. The interaction between host and its microbiota contributes fundamentally to overall health.[1] Imbalance and disruptions of the human gut microbiota in infancy and early childhood have been suggested as a risk factor for a number of lifestyle-related and immune-mediated disorders such as atopic diseases, diabetes, obesity, necrotizing enterocolitis and inflammatory bowel disease.[2–6] The neonatal period is critical in terms of mucosal defence and immunologic priming and presence of aberrant gut bacteria during this time could therefore have profound effects on immune maturation.[2,3]

A reduction in overall diversity, a reduced abundance of commensal bacteria and an increased abundance of potentially pathogenic bacteria in the gut microbiota have been associated with the development of immune-mediated disorders later in life.[2,3,7] Data from observational studies are however conflicting, exemplified by a recent study showing more diverse microbiota in children with eczema [5] and with no clear allergy-promoting or allergy-protective taxa, as opposed to another study revealing that high diversity of total microbiota and high abundance of butyrate-producing bacteria are inversely associated with severity of atopic eczema.[8] Moreover, there is ongoing debate whether in allergy development an altered diversity of the gut microbiota is more important than the altered prevalence of particular bacterial species.[9]

Representative species and strains of lactobacilli and bifidobacteria have been used as probiotics, with the aim to colonize the infant’s intestine and modulate the host’s immune response.[10] Several studies have indeed shown benefits from treatment with probiotics in atopic children and thereby modulation of the infant’s gut microbiota,[10–14] even by solely supplying mothers during late pregnancy.[15] Meta-analyses provide evidence in support of a moderate role of probiotics in the prevention of atopic dermatitis and IgE-associated atopic dermatitis in infants,[16] but more heterogeneous results are found for probiotics supplementation in the treatment of eczema.[17] Strain-specificity and the role for timing of administering probiotics are of particular importance, since some studies did not show any beneficial effect on the prevention of eczema after supplementation with probiotics.[3,5,18–21] Therefore, it seems crucial to administer probiotic supplementation during pregnancy and at least in the first months of life to be able to reasonably evaluate the effects of this intervention on microbial colonisation of the gut and possible immunological effects.

The growing recognition of the role of gut microbiota in lifelong health and disease emphasizes the need of understanding the dynamics that lead to acquisition and colonization of intestinal microbiota. Moreover, it is important to study long-term effects of probiotic supplementation, both clinically and microbiologically. The aim of this study therefore was to assess the long-term effects of added probiotics on the composition and diversity of gut microbiota over time in infants at risk for atopic disease. Moreover, we aimed to investigate the differences in microbiota between children who did and did not develop atopic disease.

## Methods

### Participants and treatment

Subjects were part of a randomized, double-blind, placebo-controlled trial (the PandA-study, registered at ClinicalTrials.gov, Identifier NCT00200954) addressing the effect of pre- and postnatal administration of selected probiotic bacteria in primary prevention of allergic disease. More detailed information about the participants, received treatments and (long-term) clinical results can be found in the supporting information (S1, S2 and S3 Files) and were described in our previous publications.[11,22] In short, all participating children had a positive family history of allergic disease (i.e. atopic eczema, food allergy, asthma or allergic rhinitis in either the mother plus an older sibling, or the father plus an older sibling with a history of allergic disease) and either received a probiotic mixture consisting of *Bifidobacterium bifidum* W23, *Bifidobacterium lactis* W52 and *Lactococcus lactis* W58 (Ecologic® Panda, Winclove Probiotics B.V., Amsterdam, the Netherlands) or placebo (the carrier of the probiotic mixture, containing rice starch and maltodextran) during their first year of life. Moreover, the probiotic mixture or placebo was prenatally administered to the mothers.

The intervention group received once daily 3 · 10^9^ colony forming units (CFU) (1 · 10^9^ CFU of each strain) of freeze dried powder of the probiotic mixture. The control group received placebo consisting of the carrier of the probiotic product, i.e. rice starch and maltodextran. Both supplements were dispensed as a stable powder in identical individually packed sachets containing 3 g of material.

After the child’s second birthday, the parents were informed about the nature of the treatment their child was exposed to. Subsequently, follow-up of the participants was continued in a single-blinded (investigator-blinded) design. When the child was approximately six years old, parents were asked to complete a follow-up visit during which clinical parameters were investigated. Parents were also asked to complete questionnaires evaluating symptoms in their child indicative of allergy.

The definition of allergy in the initial study[11] and at the age of six years[22] was made and led to the classification of ‘atopic’ versus ‘non-atopic’ in this follow-up study. Atopic children should have had at least one of the following between age 0 and 6:

- A positive doctor’s diagnosis for eczema, asthma or allergic rhinitis
- Persistent sensitization to inhalant allergens and/or food allergens (>0.35 IU/ml allergen specific IgE antibodies)

### Ethics statement

The study was approved by the Medical Ethics Committee of the University Medical Centre Utrecht, the Netherlands and has been performed in accordance with the ethical standards laid down in the 1964 Declaration of Helsinki and its later amendments. Written informed consent from the parents was obtained.

### Stool sample collection

Parents collected stool samples of their child at different time points between birth and the child’s sixth birthday. Samples of 8 time points in early life were used: one week (T1), two weeks (T2), one month (T3), three months (T4), one year (one week after the intervention was stopped) (T5), one and a half year (T6), two years (T7) and six years of age (T8).

Stool samples were collected from diapers or caught on a sheet, placed in stool collection vials and immediately frozen by the parents in their home freezers (-20°C). Frozen samples were transported on ice to the hospital at time of follow-up visits. Upon arrival, samples were immediately stored at -20°C until further analysis.

### Isolation and identification of bacteria

To isolate DNA from faeces, a pea-sized faecal sample (100–400 mg) was placed in an Eppendorf container. Then, a 200 μl suspension was made in nucliSENS lysis buffer, as provided with the easyMAG, an automated system for total nucleic acid isolation (bioMérieux Clinical Diagnostics, Marcy l'Etoile, France). This suspension was vortexed for 1 minute, shaken for 5 minutes ≥ 1400 *rpm* and subsequently centrifuged at 14,000 *rpm* for 2 minutes. Supernatant (100 μl) was transferred to an 8-welled easyMAG container, as provided by the manufacturer, and 2 ml nucliSENS lysis buffer was added. After incubation at room temperature for ≥10 min, 70 μl of magnetic silica beads was added, as provided with the easyMAG machine. Afterwards, the mixture was inserted in the easyMAG machine and the “specific A” protocol was chosen, selecting the off-board workflow and eluting DNA in 110 μl of buffer. Faecal DNA was diluted 10-fold before use in PCR. All DNA was stored at 4°C.

### 16S-23S IS profiling of gut microbiota

The technique for determination of bacterial species in faecal samples and monitoring of microbiota development over time, deviates of the description in the initial study protocol. PCR and denaturing gradient gel electrophoresis (DGGE) of 16S ribosomal DNA genes together with Fluorescent *in situ* hybridization (FISH) combined with flow cytometric analysis were performed at that time. In recent years however, the rapid development of sequencing techniques including the application of high-throughput methodology enables the analysis of hundreds of samples from different origins to conduct genetic audits of faecal material to a much greater depth than previously possible. Based on this, amplification of the interspace regions (IS-regions) was performed with the high-throughput bacterial profiling technique IS-pro (IS-diagnostics, Amsterdam, The Netherlands). This technique combines bacterial species differentiation by the length of the 16S-23S rRNA interspace region with instant taxonomic classification by phylum-specific fluorescent labeling of PCR-primers. The 16S-23S rRNA IS region is variable in size and sequence, making it well suitable for analysis of complex communities. The procedure consists of two multiplex PCRs, a combination of which provides very broad coverage for *Actinobacteria*, *Firmicutes*, *Fusobacteria and Verrucomicrobia* (AFFV), *Bacteroidetes* and *Proteobacteria*. For detailed information on the design of the used primers we refer to our previous publication.[23] Amplifications were carried out on a GeneAmp PCR system 9700 (Applied Biosystems, Foster City, CA). Cycling conditions for PCR were 94°C for 4 min; 94°C for 30 s; 35 cycles of 56°C for 45 s; 72°C for 1 min, 72°C for 11 min and a final extension at 4°C. Each PCR mixture, with a final volume of 25 μl, contained 10 μl of buffered DNA, 1x superTaq buffer (Applied Biosystems), 200 μM deoxynucleoside triphosphate, 0.04% BSA, 1 U of superTaq (Applied Biosystems), and 0.13 μM of each of the 5 primers.

### Data analysis

After pre-processing, which consisted of baseline correction and noise-filtering of data using a standardized ‘rolling ball-algorithm’ (IS-pro software suite, IS-diagnostics, Amsterdam, The Netherlands), each sample was represented by a microbial profile, consisting of color-labeled peaks. Each peak was characterized by a specific IS fragment (measured in number of nucleotides) and a color related to a specific phylum group. The intensity of peaks reflected the quantity of PCR product (measured in relative fluorescence units (RFU)). Each peak was designated as an operational taxonomic unit (OTU) and its corresponding intensity as abundance. Intensity values were log2 transformed in order to compact the range of variation in peak heights, to reduce the dominance of abundant peaks and to include less abundant species of the microbiota in downstream analyses. The cutoff level was set to <6 logRFU.[23] This transformation results in improved consistency of the estimated correlation coefficient, lower impact of inter-run variation, and improved detection of less prominent species. This conversion was used in all downstream analyses, such as calculating within-sample and between-sample microbial diversity. A clustered heat map was constructed by generating a correlation matrix based on cosine correlations of all log2 transformed profile data followed by clustering with the unweighted pair group method with arithmetic mean (UPGMA).[24]

### Diversity analysis and discriminative features selection

Diversity was calculated using the Shannon index,[25] representing diversity per time point, and differences in this index were tested with Mann-Whitney *U* test. Dissimilarities between samples, or between-sample diversity, were calculated using the cosine distance measure between each pair of samples’ profiles. Given two vectors of attributes (two profiles in our case), A and B, the cosine dissimilarity is represented using a dot product and magnitude as:

$$\text{dissimilarity}=1-\;\text{cos}\left(\theta \right)=1\;-\;\frac{\Sigma^{n}{}_{i=1}\;A_{i}\times B_{i}}{\surd \Sigma^{n}{}_{i=1}{\left(A_{i}\right)}^{2}\;\times \;\surd \Sigma^{n}{}_{i=1}{\left(B_{i}\right)}^{2}}$$

Principal coordinate analysis (PCoA), to explore similarities between groups, was calculated based on the cosine distance matrix.

Discriminative OTUs between the groups were detected by LEfSe[26] with α parameter for pairwise tests set to 0.05 and the threshold on the logarithmic score of linear discriminant analysis (LDA) set to 2.

Diversity analysis was performed using the vegan software package in R (Foundation for Statistical Computing, Vienna, Austria) and SPSS (SPSS for Mac release 22.0; SPSS Inc. Chicago, IL, USA). Differences were considered to be significant for p < 0.05.

## Results

**Fig 1:**
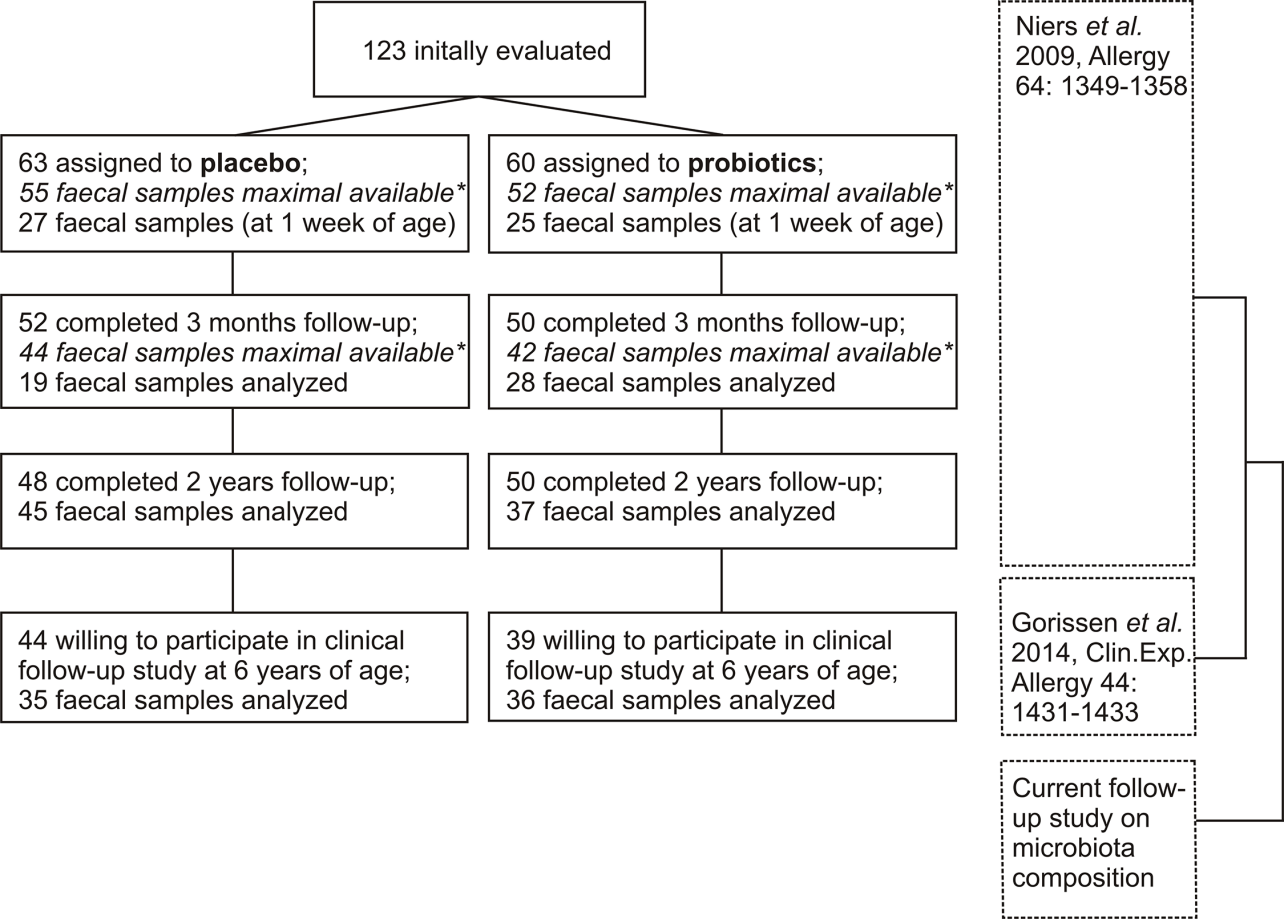
Overview of the inclusion of subjects in the initial PandA study and follow-up phases, including the number of faecal samples per time point. stool samples of eight randomly chosen participants from the placebo and intervention group at seven different time points, including those of one week and three months of age, were analyzed in the initial PandA study.

From the 123 participants evaluated in the initial PandA-study, 108 fully completed the initial prenatal intervention and 98 fully completed the initial postnatal intervention (until two years of age). 102 consented to be contacted for follow-up and a total of 83 participants were willing to participate in the follow-up study at the age of six years. Reasons for not willing to participate primarily were lack of time or priority. Clinical data of this follow-up study have been published previously.[22] In short, administration of the selected combination of probiotics did demonstrate a beneficial effect on the development of eczema up to the age of two years. This preventive effect was established within the first 3 months of life (12% parental reported eczema in the probiotics group vs 29% in the placebo group).[11] No differences were observed in respiratory symptoms indicative for asthma or allergic rhinitis at the age of 2 years. The beneficial effect on development of eczema did not extend to the age of 6 years and did not lead to primary prevention of asthma.[22] Comparison of baseline characteristics of the initial group to those of this follow-up group did not indicate selection bias due to selective drop-out. In total, of 99 children a stool sample at one or more different time points during the complete study period could be collected (until six years of age). A flow diagram of the initial clinical trial and follow-up studies is represented in (Fig 1 **)**. It also shows how this follow-up study relates to the previous studies. Relatively low percentages of subjects with faecal samples at early time points are due to the hectic postpartum period (one week of age) and previous analyses (in the original PandA study) that decreased the number of samples maximal available for the first five time points. The distribution of number of samples per time point, subdivided in probiotic/placebo and atopic/non-atopic groups, are shown in Table 1 **.**

**Table 1:** Number of samples per time point per group (n, %).

	Placebo		Probiotic	
Time point	Atopic	Non-atopic	Atopic	Non-atopic
**(T1) One week, *n = 52***	16 (30.8%)	11 (21.2%)	15 (28.8%)	10 (19.2%)
**(T2) Two weeks, *n = 39***	9 (23.2%)	10 (25.6%)	10 (25.6%)	10 (25.6%)
**(T3) One month, *n = 42***	9 (21.4%)	8 (19.1%)	14 (33.3%)	11 (26.2%)
**(T4) Three months, *n = 47***	11 (23.4%)	8 (17.0%)	19 (40.5%)	9 (19.1%)
**(T5) One year, *n = 63***	17 (27.0%)	13 (20.6%)	21 (33.4%)	12 (19.0%)
**(T6) One and a half year, *n = 69***	23 (33.3%)	13 (18.9%)	22 (31.9%)	11 (15.9%)
**(T7) Two years, *n = 82***	30 (36.6%)	15 (18.3%)	26 (31.7%)	11 (13.4%)
**(T8) Six years, *n = 71***	24 (33.8%)	11 (15.5%)	24 (33.8%)	12 (16.9%)

T = time point

### Gut microbiota migration over time

**Fig 2:**
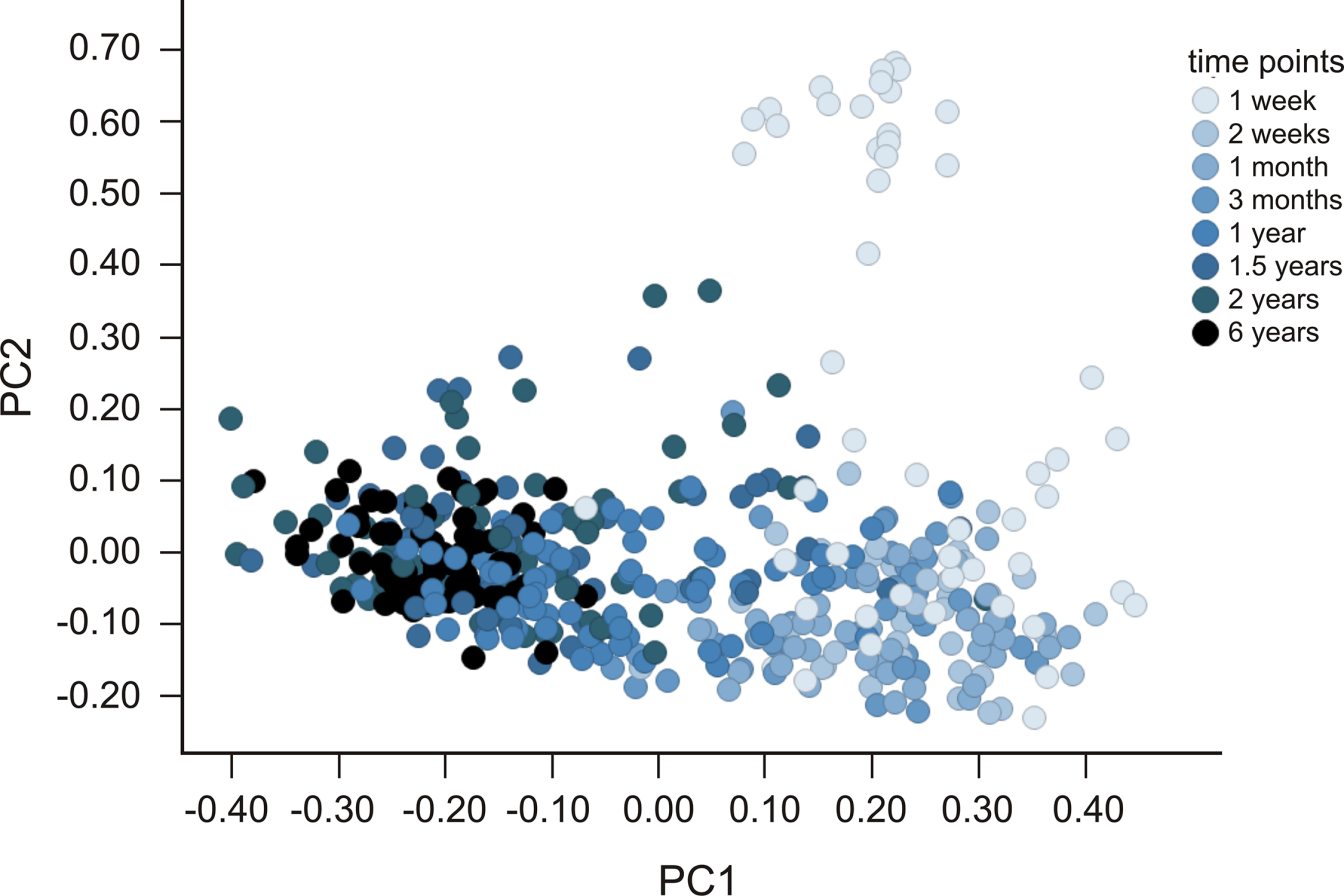
Principle coordinate analysis (PCoA) scatterplot to express gut microbiota development over time (T1-T8), all phyla combined, in the study population. Smaller inter-individual distances indicate more similar microbiota composition. Colour intensity (light to dark) reflects the age of a child at which the sample was taken.

**Fig 3:**
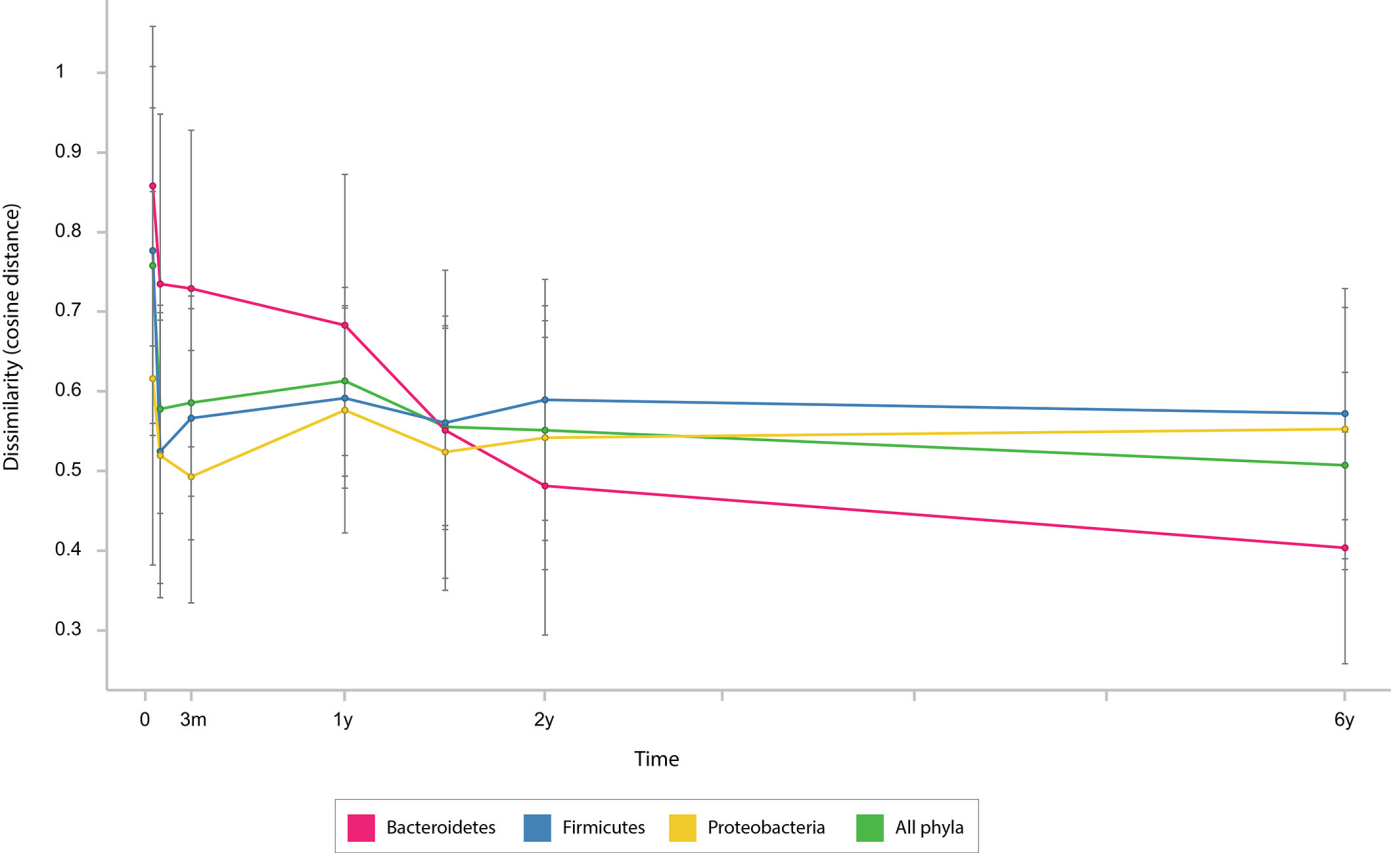
Microbiota stability over time. Depicted are average cosine distances (y-axis) between two subsequent time points (x-axis) for each phylum and all phyla combined.

First, we looked for general features of the development of gut microbiota composition between 0 and 6 years of age. The microbiota development was highly associated with age, as shown in Fig 2. Samples are most heterogeneous at early age and from there follow a more or less conserved vector towards a fairly homogeneous cluster at the age of six. Stability of the microbial community on phylum level was investigated by calculating average cosine distances for each phylum between two subsequent time points. Fig 3 gives a depiction of community stability over time and underlines that the largest variations in microbiota composition occur very early in life. Moreover, it can be seen that *Bacteroidetes* populations stabilize between one and two years of age, and variations clearly decrease from two years of age.

### Effect of probiotic supplementation on gut microbiota composition

#### Probiotic versus placebo group

Secondly, we investigated whether the probiotic product, containing *Bifidobacterium bifidum* W23, *Bifidobacterium lactis* W52, and *Lactococcus lactis* W58 could be detected in the faecal samples. Indeed, the presence of the supplied product was confirmed. IS-pro could not discriminate between the two *Bifidobacterium* strains, since both gave a partially overlapping microbial profile.

**Fig 4:**
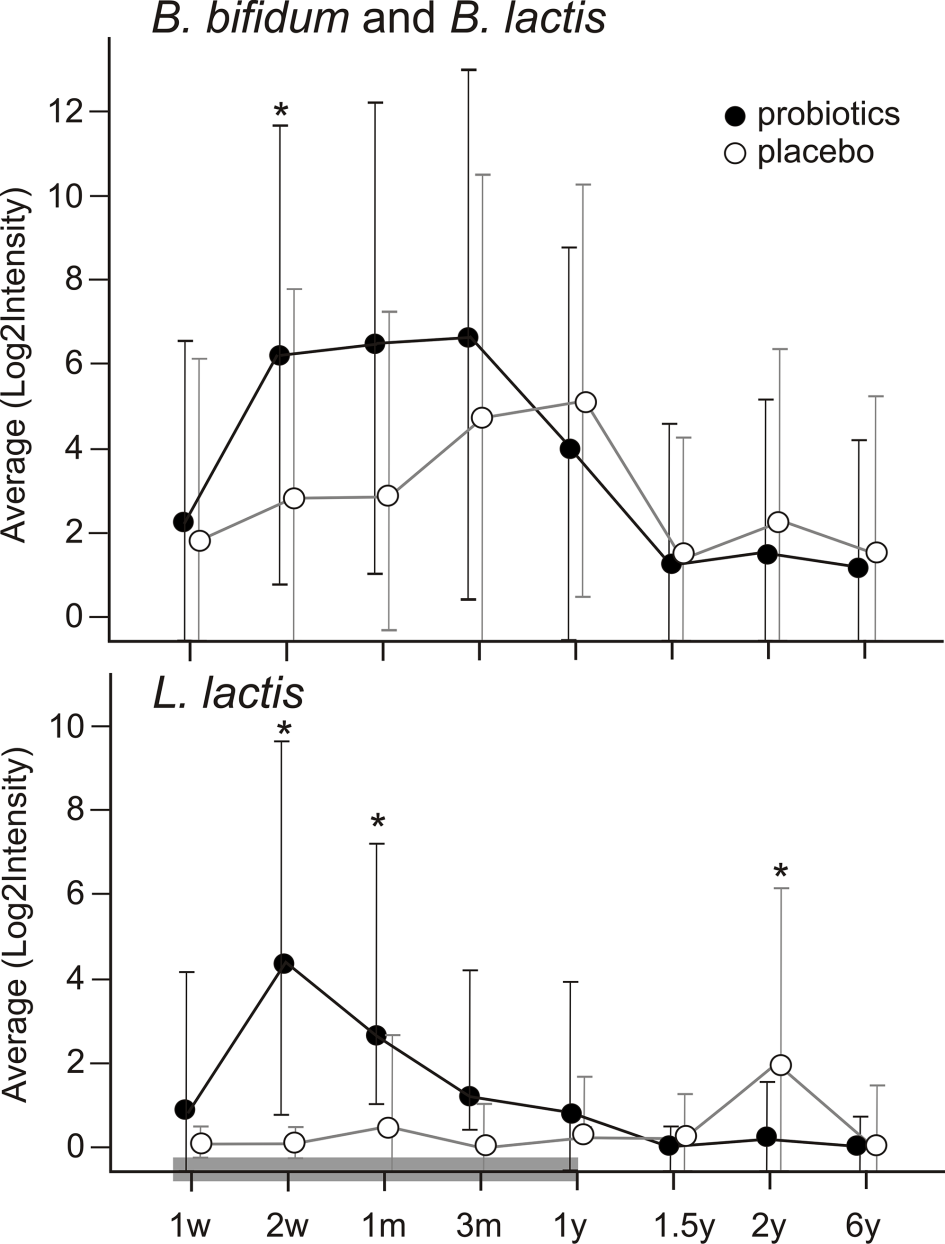
Relative abundance (y-axis) per time point (x-axis) of the probiotic strains B. Bifidum/B. Lactis and L. Lactis (dots) and error bars. Marked bar on the x-axis indicates intervention period. * = significant different time points.

At time of supplementation (T1-T5) the probiotic species had a higher abundance and prevalence in the probiotic group, whilst after stopping (T6-T8) prevalence was comparable between the two groups. Fig 4 shows the relative abundances per time point for the *Bifidobacterium* strains and *Lactococcus* strain. Bifidobacteria were significantly higher in the probiotic group at one month of age (T3, p = 0.003) and *Lactococcus lactis* was significantly higher at two weeks of age (T2, p = 0.001), and one month of age (T3, p = 0.03). Moreover, *Lactococcus lactis* was substantially absent in the placebo group during the intervention period and had a significantly higher abundance at the age of two years in this group (T7, p = 0.01).

Subsequently, we searched for potential effects of the intervention on microbiota composition at the species or genus level. By using LEfSe,[26] a method designed to explain differences between microbial communities, no bacterial species or genus that were significantly different between the treatment groups could be identified.

**Fig 5:**
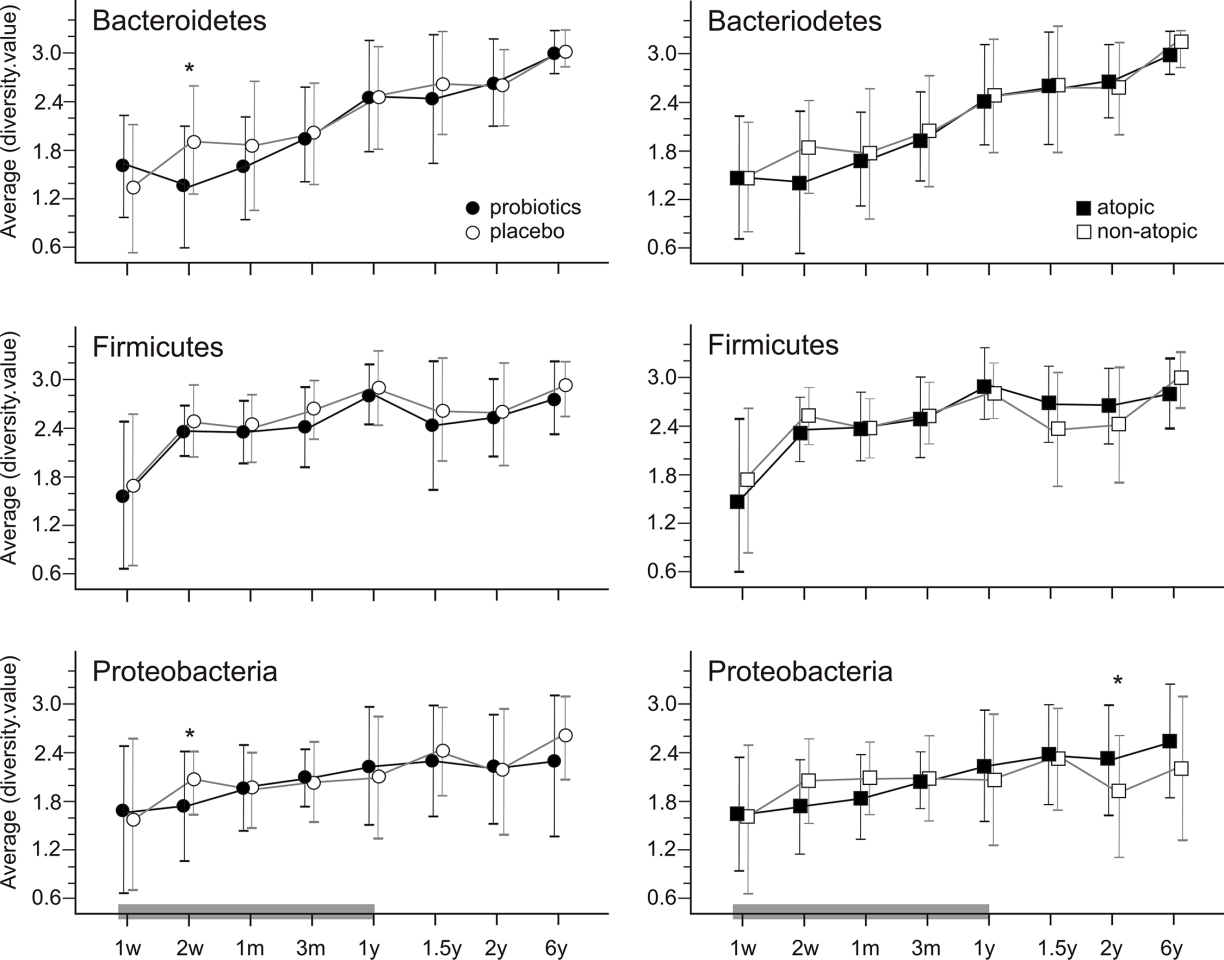
Diversity index (Shannon, y-axis) of the phyla *Bacteroidetes*, *Firmicutes* and *Proteobacteria* per time point (x-axis) for probiotics versus placebo group (A) and atopic versus non-atopic group (B) (dots, with error bars). Marked bar on the x-axis indicates intervention period. * = significant different time points.

At phylum level, there were no major differences between the probiotic and placebo group according to microbiota diversity (of *Bacteroidetes*, *Firmicutes* and *Proteobacteria*), except at the age of two weeks where the diversity of Bacteroidetes and Proteobacteria in the placebo group was higher (T2, p< 0.05) compared to the probiotic group (Fig 5A). Relative abundances per phylum in the probiotic group and placebo group, that show the similar pattern compared with diversity, are shown in S1 Fig.

#### Atopic versus non-atopic group

As described above, no major differences between the probiotic and placebo group could be identified according to microbiota diversity. The design of the study also allowed us to study potential differences in gut microbiota development in children who did and did not develop atopy in early life. Fig 5B shows diversity per phylum in the atopic and non-atopic groups. Diversity of all three phyla increased over time and no significant differences were found between the atopic and non-atopic group, except at the age of two years when atopic children had a significantly higher *Proteobacteria* diversity (T7, p< 0.001, Mann-Whitney U-test).

The relative abundance of the phyla *Firmicutes* and *Bacteroidetes* generally increased over time and followed a similar trend at all time points in the atopic and non-atopic group; no statistical differences were found (S1 Fig). As *Proteobacteria* are amplified in a separate PCR reaction, their relative abundance could not be quantified together with the other two phyla. But in accordance with *Bacteroidetes* and *Firmicutes*, relative abundance of *Proteobacteria* increased over time.

At species or genus level, differences between the microbial community in the atopic and non-atopic group was tested using LEfSe,[26] but no discriminative bacterial species that were significantly different between the disease groups, could be identified.

### Effects of probiotic intervention or disease status over age

**Fig 6:**
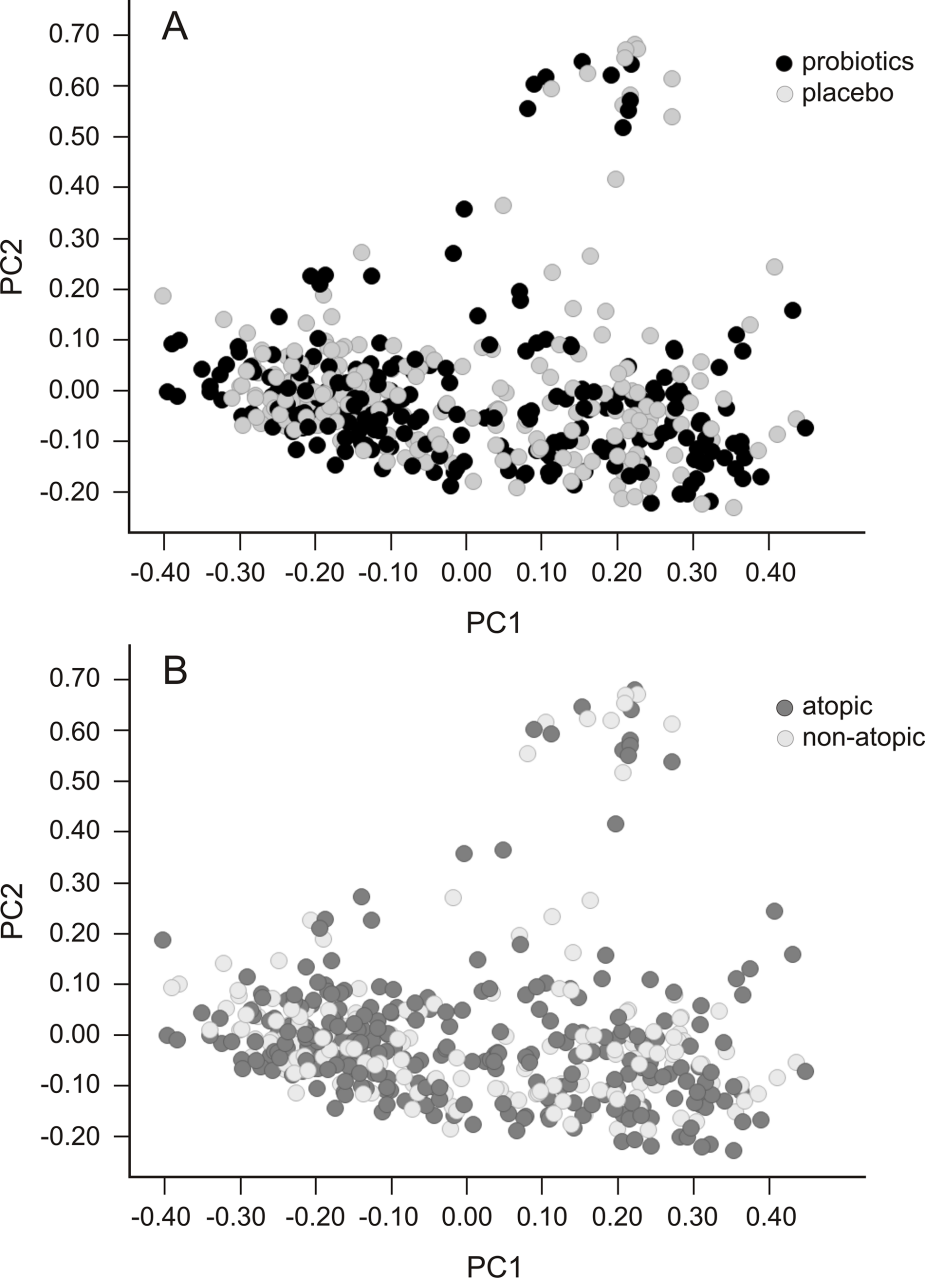
(Principle coordinate analysis) PCoA representing gut microbiota development. Samples are grouped by intervention (A) and disease (B). Dots represent microbiota composition (all phyla combined) per individual per time point.

Finally, we investigated the development of microbiota composition over age depending on the intervention or a child’s atopic disease status. A similar development of a stable, robust microbiota composition over time was found in all subgroups, and therefore was not associated with later development of atopic disease or influenced by supplementation with probiotics (Fig 6).

## Discussion and Conclusion

In this study the long-term development of gut microbiota was investigated in infants at high-risk for atopic disease, who were supplemented with probiotics or placebo during the first year of life. Follow-up ended at the age of six years. Our data suggest that, regardless of intervention or atopic disease status, children develop a stable, converging gut microbiota during their first years of life. Age was the major driver of microbiota composition, overriding the differences based on intervention or development of atopic disease and the gut microbiome development is suggested to continue between 2 and 6 years of age.

In line with this finding,intestinal microbiota development is currently thought to stabilize by the end of the first year of life and the composition considered to resemble the adult gastrointestinal tract by the age of 2 to 2.5 years.[6,27–29] Koenig *et al*.[29] showed that the qualitative measures of diversity responded to time, but the quantitative measures (such as abundance) responded to life events (illnesses, dietary changes, antibiotic treatment). Yatsunenko *et al*. [30] described an adult-like configuration within a three-year period after birth and showed some differences in clustering between very young children and adults. Results of Ringel-Kulka *et al*. [4] extend this difference (by describing significant differences in diversity between the group of 3–4 years old children and adults) and indicate that the microbiota has not yet reached the climax of bacterial diversity at the age of 4 years. The authors suggest that the evolvement into adult-like microbiota continues beyond 3 years of age. Our data support the last suggestion as the gut microbiome development seemed to continue between 2 and 6 years of age, though a specific time point for microbiome stabilization could not be indicated.

The presence of probiotic strains in stool samples was confirmed during supplementation. The fact that *Lactococcus lactis* was substantially absent in the placebo group during the intervention period is not surprising as the *Lactococcus* genus is not considered a commensal in infants. There was however a significantly higher presence and abundance of *Lactococcus lactis* at the age of two years (T7) in the placebo group compared to the probiotic group (9/45 versus 1/37) as we specifically analysed the presence of this strain being part of the supplied probiotic product. This difference can be caused by the introduction of solid food and the variability of the children’s diet at this age, because *Lactococcus lactis* is a well-known ingredient of buttermilk and cheese. When analysing the prevalence of *Lc*. *lactis* at the age of two years in more detail, there was a tendency towards a higher prevalence in the atopic children within the placebo group (7/45) when compared to the non-atopic ones (2/45). This difference was however not significant after correction for multiple testing.

At the age of two weeks (T2) the diversity of Bacteroidetes was significantly higher in the placebo group compared to the probiotic group, a finding also described by Enomoto *et al*.[14] at the age of four months. This could suggest that probiotics suppress the acquisition of genera belonging to the phylum Bacteroidetes. Hypothetically, this could cause relevant disturbances on gut microbial patterns over time and have effects on clinical outcomes, since lower diversity of the phylum Bacteroidetes has been related to increased risk of asthma and atopic sensitization.[5,31] However, clinical results of the present study showed significantly lower eczema during the first 3 months of life in the intervention group compared with placebo, which does not point towards a predisposing role for the development of asthma and atopy in our population. Moreover, the difference demonstrated here seems to be without long-term consequences as microbiota composition of placebo and probiotic children showed no significant differences anymore during follow-up.

In this study no evident long-term consequences of probiotic supplementation on microbiota composition were found, as the abundance of probiotic strains decreased after the intervention and effects on diversity and abundance disappeared afterwards. Our results are in line with those described by Nylund *et al*.[5] who showed that *Lactobacillus rhamnosus* GG supplementation during the first 6 months of life, in children with and without eczema, had only minor long-term effect on the microbiota composition. Enomoto *et al*.[14] also demonstrated limited changes in the composition of faecal microbiota after bifidobacteria supplementation during the first six months of life to healthy infants.

The exact in vivo mechanism of action of probiotics in shaping the immune response still needs to be determined and a number of unanswered questions remain regarding how probiotics mediate their clinical effects. The intestinal microbiome may contribute to the pathogenesis of allergic diseases due to its substantial effect on mucosal immunity. A probiotic may have a direct interaction with the ecosystem within the gut lumen by providing enzymatic activities that cause metabolic effects, it can interact with the gut lumen mucus and epithelium, and may be signaling the host to other organs beyond the gut.[32]

The design of the present study allowed us also to study potential differences in gut microbiota development in children who did and did not develop atopy. Variations in early gut colonization have been associated with the development of atopic disease. Results have, however, been highly variable across studies. Wang *et al*.[7] examined overall patterns of faecal microbial colonization in healthy and atopic infants and found that infants who developed eczema had significantly lower faecal bacterial diversity at 1 week of age. Others have confirmed that reduced gut microbial diversity in early life was associated with an increased risk of eczema[9,33] and multiple differences in specific bacterial groups result in microbiota profiles that are significantly distinct between healthy and eczematous infants.[5]

Johansson *et al*. showed that the kinetics of colonization postnatally seemed to differ generally in non-allergic children compared to allergic children, with a delayed colonization in early infancy in the allergic group. At 12 months of age however, the groups of children were similar in the frequencies of the different species investigated. Bisgaard *et al*.[2] described a population of high-risk infants comparable to the present study and showed an association between reduced bacterial diversity of the infant’s intestinal microbiota with increased risk of allergic sensitization, allergic rhinitis and peripheral blood eosinophilia, but not asthma or atopic dermatitis, in the first 6 years of life.

In contrast to the above findings, in our study, abundance and diversity were the same over time in atopic and non-atopic children though small differences were seen. This is in accordance with a prospective study from 3 European birth cohorts that also found no differences in gut microbiota among infants developing or not developing atopic eczema and food allergy.[34]

Differences in findings between all these studies, including ours, may reflect differences in methods used for investigating the faecal microbiota or even differences in defining atopy. Our study does endorse the importance of the gut microbiota composition in early life, because of the beneficial clinical effect of probiotics on eczema,[11] but besides effects on gut microbiota composition during the intervention, no long lasting differences could be identified. In this respect, recent data point towards a major role for short chain fatty acids production, especially butyrate, in regulation of development of mucosal regulatory T cells. Therefore, next to the 16S-23S rRNA gene analysis, as performed in this study, microbial metagenomics (whole genome shotgun sequencing) could further expand the understanding of gut microbiota development and composition and microbial metabolomics may reveal mechanisms by which gut microbiota interacts with the human host.[35,36]

In conclusion, supplementation with a probiotic mixture consisting of *B*. *bifidum* W23, *B*. *lactis* W52 and *Lc*. *Lactis* W58 to children at high-risk for atopic disease had only minor effects on gut microbiota composition. Future studies should identify the functional activities of the gut microbiota, but also further elucidate the working mechanisms of probiotics, to illustrate host and microbiota interactions and identify optimal timing and duration of probiotic supplementation as strategy for prevention of allergic diseases.

## Supporting Information

S1 Fig Average abundances of the phyla *Bacteroidetes*, *Firmicutes* and *Proteobacteria* per time point (x-axis) for probiotics versus placebo group and atopic versus non-atopic group (dots/squares, with error bars). (EPS)

S1 File Study protocol PandA study (Dutch). (PDF)

S2 File English summary study protocol PandA study. (PDF)

S3 File Study protocol PandA follow-up study. (PDF)
