# Supplementary material for: Long Term Development of Gut Microbiota Composition in Atopic Children: Impact of Probiotics
Source: PLoS One. 2015 Sep 17;10(9):e0137681. doi: 10.1371/journal.pone.0137681 (PMC4574972; doi:10.1371/journal.pone.0137681)
Supplement: S1 File — (PDF) [file pone.0137681.s002.pdf]

PANDA STUDIE

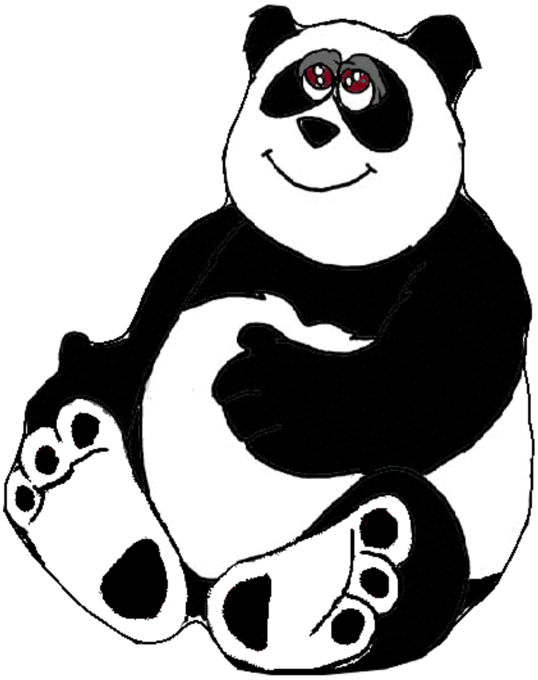

Probiotics AND Allergie

Primaire preventie van astma en allergie
door perinatale toediening van probiotica

Onderzoeksprotocol, versie 4, Utrecht januari 2004

Inhoudsopgave

1. Samenvatting 3
2. Introductie en vraagstelling 5
3. Belang van dit onderzoek voor de volksgezondheid 7
4. Studieopzet
    4.1 Tijdschema 8
    4.2 Statistiek 8
    4.3 Patiënten 8
    4.4 Voeding 9
    4.5 Methoden en uitkomstvariabelen
        4.5.1. Uitkomstvariabelen Hoofdvraagstelling A 9
        4.5.2. Uitkomstvariabelen Afgeleide vraagstelling B 11
        4.5.3. Uitkomstvariabelen Afgeleide vraagstelling C 12
        4.5.4. Uitkomstvariabelen Afgeleide vraagstelling D 13
5. Probiotica preparaat 14
6. Vooronderzoek 16
7. Onderzoekslijnen UMCU waar PANDA op aansluit 17
8. Ethische overwegingen 18
9. Organisatie 20
10. Literatuur 21

Bijlagen:

I Vragenlijst PANDA studie 23
II Patiënten Informatiebrief 27
III Informed Consent 30
IV Verzekering 31
V Serious adverse Events formulier 33

1. Samenvatting

Achtergrond

Allergie komt in toenemende mate voor in Westerse landen. De hygiëne hypothese suggereert dat de toename van allergische aandoeningen het gevolg is van een verminderde blootstelling aan microbiële antigenen. Daarnaast staat algemeen vast dat de immunologische basis voor het ontwikkelen van allergie reeds op zeer jonge leeftijd wordt gelegd. Voor succesvol ingrijpen in de immuunrespons van de zuigeling bestaat een zogenaamde “window of opportunity” die gelegen is in de eerste 6 levensmaanden. Indien getracht wordt het immuunsysteem te moduleren door in te grijpen in de kolonisatie van de darm dan dient dit in de eerste levensweek te gebeuren. Als mogelijke nieuwe preventieve therapie voor het ontwikkelen van allergie is het gebruik van probiotica gesuggereerd. Probiotica zijn microben die aanwezig zijn in de humane intestinale flora en een immuunrespons kunnen induceren. Probiotica, waaronder melkzuurbacteriën komen significant meer voor in de darmflora van niet-allergische kinderen ten opzichte van allergische kinderen. Probiotica toegediend aan zwangere vrouwen met allergie in de laatste 6 weken van de zwangerschap en aan hun pasgeborenen tot de leeftijd van 6 maanden resulteerde in een reductie van de prevalentie van eczeem met bijna 50 %. Bovenstaande gegevens en observaties suggereren dat vroege toediening van probiotica aan zuigelingen met een hoog risico op het ontwikkelen van allergie kan leiden tot preventie van allergie. Desondanks zijn meerdere belangrijke vragen nog onbeantwoord waaronder: wat is het werkingsmechanisme van probiotica, wat is het immuunmodulerend potentieel van andere (combinaties van) probiotica?

Doel van de studie

Het voorkomen of verminderen van klachten van astma, (atopisch) eczeem, hooikoorts en (voedsel-)allergie bij kinderen met een hoog risico op het ontwikkelen hiervan door middel van perinatale toediening van probiotica

Vraagstelling

<u>Hoofdvraagstelling A</u>: wat is het effect van vroege toediening van probiotica op astma, (atopisch) eczeem, hooikoorts en (voedsel-)allergie op de kinderleeftijd?

<u>Afgeleide vraagstellingen:</u>

<u>B</u>: Leidt vroege toediening van probiotica tot een blijvende andere samenstelling van de darmflora ten opzichte van de placebogroep?

<u>C</u>: Geeft vroege toediening van probiotica in-vivo modulatie van het immuunsysteem? (Suppressie van Thelper-2-cel respons of inductie van regulatoire T cellen?)

<u>D</u>: Spelen genetische polymorfismen van moleculen en receptoren van het immuunsysteem een bepalende rol in de respons op probiotica (“gene by environment interaction”)?

Werkplan / methode

Dubbelblind gerandomiseerd placebo gecontroleerde studie naar klinische effecten en immunomodulatie van probiotica in zwangere vrouwen die bekend zijn met atopische ziekte en hun pasgeborenen. Zwangere vrouwen gebruiken de probiotica in de laatste 8 weken van de zwangerschap en vervolgens de pasgeborenen tot de leeftijd van 1 jaar. De klinische follow-up is tot de leeftijd van 2 jaar met als primaire uitkomstvariabele de incidentie van allergische aandoeningen. Onderzoek naar de effecten op het

immuunsysteem als secundaire uitkomstvariabele worden afgemeten aan productie van cytokinen en morfologie van mononucleaire cellen uit perifeer bloed. De keuze voor een bepaalde probiotische stam in de klinische trial is gebaseerd op de resultaten van de in-vitro studie en van het diermodel.

Deelnemers

- *Inclusie:*

Zwangere vrouwen die in heden of verleden bekend zijn met of door arts gediagnosticeerd zijn met allergische aandoeningen als astma, allergische rhinitis (hooikoorts), voedselallergie, atopisch eczeem. Of gezinnen waarin zowel de vader als één of meer van de oudere kinderen is aangedaan met één van de beschreven aandoeningen.

Elke medicamenteuze behandeling voorgeschreven door een arts is toegestaan maar dient te worden gemeld aan de onderzoekers.

- *Exclusie:*

Het voornaamste exclusie criterium is het niet kunnen starten van de interventie / placebo bij de neonaat of indien de interventie beïnvloed wordt door antibiotica gebruik. Antenataal systemisch gebruik van medicijnen die het afweersysteem beïnvloeden, als corticosteroïden zijn een exclusie criteria. Neonataal glucocorticoïde therapie geldt als exclusie criterium. Het gebruik van regulier gebruik van probiotica-houdende producten is tevens een exclusie criterium.

Belasting en risico proefpersonen

Het toedienen van probiotica aan volwassenen en kinderen wordt als veilig beschouwd. Daarnaast wordt er in deze studie gebruik gemaakt van probiotische stammen die reeds veelvuldig gebruikt worden in voedingssupplementen en zuivelproducten. Met name de pasgeborene wordt in dit onderzoek belast met op 3 tijdstippen een venapunctie en op de leeftijd van 2 jaar huidtesten voor allergie. Huidtesten worden door kinderen van deze leeftijd i.h.a. niet als vervelend ervaren en de belasting van een venapunctie wordt zoveel mogelijk verminderd door de huid van tevoren te verdoven.

Probiotica product

Op basis van uitgebreid in-vitro onderzoek zijn in samenwerking met Winclove Bioindustries ®, Amsterdam, 3 stammen gekozen die als multi-species preparaat zullen worden toegediend in een hypoallergeen drager (rijstzetmeel) in flesjes van 10 ml partieel gehydrolyseerde melk in een dagelijkse dosis van 3×10^9 CFUs. De placebo behandeling bestaat uit de partieel gehydrolyseerde melk met drager. Het staat de ouders / deelnemers vrij om te kiezen tussen borstvoeding of flesvoeding.

Eindpunten

Primaire eindpunt is de incidentie en prevalentie van astma, (atopisch) eczeem, hooikoorts en (voedsel-)allergie op de leeftijd van 2 jaar.

2. Introductie en Vraagstelling

Gedurende de laatste decennia is er een forse toename van allergie en allergische aandoeningen met name onder kinderen. De huidige behandelingsmogelijkheden geven geen blijvende verbetering. In deze studie zal met perinatale toediening van probiotica de mogelijkheden van primaire preventie van allergische aandoeningen onderzocht worden.

Onze hypothese is dat vroege toediening van probiotica aan zwangere vrouwen met allergie en vervolgens aan hun pasgeborenen leidt tot het voorkomen of verminderde ontwikkeling van sensibilisatie en allergische aandoeningen bij het kind.

Deze hypothese is gebaseerd op de volgende waarnemingen:
Allergie wordt immunologisch gekenmerkt door overexpressie van type 2 T helper (Th2) cel immuunresponsen die al vroeg in het leven aanwezig zijn.[6-8] Opvallend is dat het immuunsysteem van een foetus en pasgeborene reeds Th2 dominant is. Uitgebalanceerde rijping van het immuunsysteem dient tot stand te komen door postnatale blootstelling aan microbiële antigenen die bij voorkeur leiden tot type 1 T helper (Th1) cel immuunresponsen. Indien het Th2 dominante immuunsysteem van de pasgeborene in stand wordt gehouden door verminderde blootstelling aan microbiële antigenen kan dit leiden tot allergische aandoeningen. De hygiëne hypothese suggereert dan ook dat de toename van allergie kan worden toegeschreven aan een verminderde blootstelling aan microbiële antigenen op de kinderleeftijd.[1-3] Gezinsgrootte, verbeterde hygiënische omstandigheden, vaccinaties en het gebruik van antibiotica kunnen volgens deze hypothese een belangrijke rol spelen in de verminderde blootstelling aan microbiële antigenen.[4, 5] Een essentiële rol in dat proces lijkt te zijn weggelegd voor de kolonisatie en opbouw van de darmflora van de pasgeborene. Aangezien de darmmucosa het grootste oppervlak is van het menselijk lichaam waar direct contact plaats vindt tussen antigenen en de gastheer lijkt de opbouw van de darmflora belangrijk te zijn voor een evenwichtige uitrijping van het neonatale immuunsysteem en de ontwikkeling van tolerantie.[15] De rol van de darmflora in de ontwikkeling van allergie wordt geïllustreerd aan de verschillen tussen de darmflora van kinderen in de ontwikkelde landen (met een hoge prevalentie van allergie) en ontwikkelingslanden waar bifidobacteria en lactobacilli de initiële darmflora domineren. Vergelijking van de darmflora van allergische en niet-allergische kinderen suggereerde eveneens dat allergische kinderen in mindere mate gekoloniseerd waren met lactobacilli en bifidobacteria.[13, 14] Lactobacilli en bifidobacteria staan ook wel bekend als probiotica. In in-vitro studies is aangetoond dat probiotica invloed hebben op productie van cytokinen van mononucleaire cellen. Probiotica bewerkstellingen een verschuiving naar een Th1-respons, onderdrukking van Th2-respons en mogelijk inductie van een regulatoire immuunrespons [20-25], hetgeen uitzicht biedt op therapeutische toepassingen voor patiënten met Th2-gerelateerde ziektes als allergie en astma. Het aantal klinische studies met probiotica toepast bij allergische aandoeningen is gering. De meerderheid zijn tertiaire preventie studies [28-34], waarvan de resultaten niet eensluidend zijn en niet voldoende overtuigend zijn. In de vooralsnog enige primaire preventie studie werd een duidelijk gunstig effect gezien van probiotica op de ontwikkeling van eczeem. Er was geen effect op het ontstaan van atopie, gemeten als een verhoogde serum IgE concentratie.[26, 27]

Bovenstaande intrigerende gegevens en observaties suggereren dat vroege toediening van probiotica aan zuigelingen met een hoog risico op het ontwikkelen van allergie kan leiden tot preventie van allergie en vermindering van de morbiditeit van allergische aandoeningen.
Desondanks zijn meerdere belangrijke vragen nog onbeantwoord waaronder: wat is het werkingsmechanisme van probiotica, wat zijn de mogelijkheden van andere (combinaties van) probiotica, wat is het belang van reeds prenatale toediening van probiotica? Daarnaast dienen de resultaten van recentelijk verschenen studies bevestigd te worden alvorens definitieve conclusies over de toepassing van probiotica bij allergische aandoeningen getrokken kunnen worden.
In de beschreven studie willen wij dan ook antwoord geven op de volgende vragen:
Hoofdvraag A: Wat is het effect van vroege toediening van probiotica op allergische aandoeningen op de kinderleeftijd (i.e. atopische dermatitis, koemelkeiwitallergie).
Afgeleide vragen:
B: Leidt vroege toediening van probiotica tot een blijvende andere samenstelling van de darmflora ten opzichte van de placebogroep?
C: Geeft vroege toediening van probiotica in-vivo modulatie van het immuunsysteem? (suppressie van de Thelper-2-cel respons of inductie van regulatoire T cellen?)
D: Spelen genetische polymorfismen van moleculen en receptoren van het immuunsysteem een bepalende rol in de respons op probiotica ("gene by environment interaction")?

Samengevat luidt de vraagstelling van dit onderzoeksproject: leidt vroege toediening van probiotica tot verminderde ontwikkeling van allergische aandoeningen en kan dit effect gerelateerd worden aan een andere samenstelling van de darmflora en modulatie van immuunresponsen?

3. Belang van dit onderzoek voor de volksgezondheid

Allergie komt in toenemende mate voor in Westerse landen. Al eerder waarschuwde de WHO voor de epidemische omvang van allergieën. Volgens de WHO is er sprake van een verdubbeling sinds de jaren zeventig. Deze toename treft voornamelijk kinderen. In de internationale literatuur wordt geschat dat 30 % van de kinderen tot 18 jaar last heeft van allergie of allergische aandoeningen. In Nederland gelden ongeveer dezelfde getallen. Hiermee zijn allergische aandoeningen dus verantwoordelijk voor een groot deel van de morbiditeit op de kinderleeftijd. Allergie is een multiorgaanziekte met een breed scala aan klachten als jeuk, eczeem, huiduitslag, gastrointestinale problemen en in het meest ernstige geval anafylactische shock. Tot op heden richt de behandeling van allergische aandoeningen zich voornamelijk op het beperken of behandelen van klachten. Bijvoorbeeld door gebruik van medicijnen tijdens of ter voorkoming van klachten en vermijden van contacten met allergenen door voedingsadviezen en eliminatie diëten, geen huisdieren en saneren van het huis. De ontwikkeling van nieuwe mogelijkheden voor primaire preventie van allergie is daarom van groot belang voor de volksgezondheid. Probiotica zijn hiervoor een veelbelovende kandidaat. De beschreven klinische interventie studie beoogt meer inzicht geven in de effecten van toediening van probiotica op de ontwikkeling van allergische aandoeningen op de kinderleeftijd. Daarnaast zal deze studie de rol van de darmflora en de effecten op het immuunsysteem verduidelijken.

4. Studie opzet

Om onze hypothese te toetsen en onze vraagstellingen te kunnen beantwoorden is gekozen voor een gerandomiseerde, dubbel blind, placebo gecontroleerde klinische studie in zwangere vrouwen bekend met allergische aandoeningen en hun hoog-risico kinderen. De probiotica of placebo worden dagelijks gegeven vanaf 4-6 weken voor de geboorte. Na de geboorte krijgen de pasgeborenen probiotica of placebo (afhankelijk van de randomisatie van moeder) gedurende hun eerste levensjaar. De follow-up is tot de leeftijd van 2 jaar.

4.1 Tijdschema

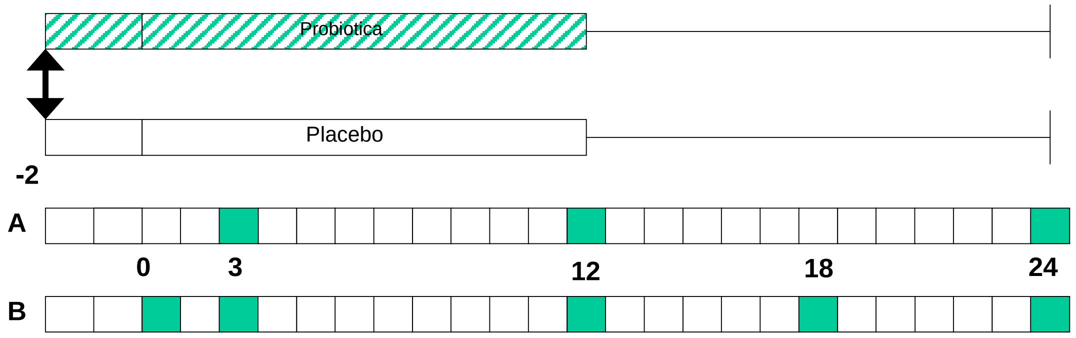

Tijd aangeduid in maanden

0 = tijdstip geboorte en afname navelstrengbloed

Randomisatie op tijdstip -2 maanden

A = follow-up bezoeken + afname perifeer bloed, bij 24 maanden inclusief huidpriktesten voor allergie

B = tijdstip van afname faeces monsters

4.2 Statistiek

Voor de power analyse is uitgegaan van een 25% reductie in de cumulatieve prevalentie van atopisch eczeem in de interventie groep. Dit betekent een aantal van 50 kinderen per groep. Ervan uitgaande dat 20% van de deelnemers na inclusie hun medewerking opzeggen, zullen 120 zwangere vrouwen met allergische aandoeningen moeten worden geïncludeerd. Voor verwerking van de data zal gebruik worden gemaakt van SPSS. De data zullen worden gestratificeerd naar maternaal roken, maternaal eczeem en voeding van het kind.

4.3 Patiënten

Inclusie:

Zwangere vrouwen die in heden of verleden bekend zijn met of door arts gediagnosticeerd zijn met allergische aandoeningen als astma, allergische rhinitis (hooikoorts), voedselallergie, atopisch eczeem. Of gezinnen waarin zowel de vader als één of meer van de oudere kinderen is aangedaan met één van de beschreven aandoeningen.

Gezinnen met een ouder kind met koemelkeiwit allergie zullen niet worden geïncludeerd aangezien het in Nederland beleid is volgende pasgeborenen in het gezin gehydrolyseerde voeding te geven.
Elke medicamenteuze behandeling voorgeschreven door een arts is toegestaan maar dient te worden gemeld aan de onderzoekers.

Exclusie:
Het voornaamste exclusie criterium is het niet kunnen starten van de interventie / placebo bij de neonaat of indien de interventie beïnvloed wordt door antibiotica gebruik. In de praktijk betekent die exclusie van prematuur (< 37 W AD) geboren kinderen die opname behoeven en a terme neonaten die in verband met infectie opgenomen worden voor antibiotica. Bij beide groepen neonaten zal de introductie en start van de voeding anders verlopen dan normaal in de thuissituatie zou hebben plaats gevonden, wat de start van de interventie / placebo vertraagd en bemoeilijkt, zoniet onmogelijk maakt. Aangezien de moeder de laatste 6 weken van de zwangerschap ook probiotica / placebo gebruikt zal antibiotica gebruik 2 weken voor de bevalling exclusie betekenen van de betreffende deelnemer.
Antenataal systemisch gebruik van medicijnen die het afweersysteem beïnvloeden, als corticosteroïden zijn een exclusie criteria. Neonataal glucocorticoïde therapie geldt als exclusie criterium. Het gebruik van regulier gebruik van probiotica-houdende producten is tevens een exclusie criterium.

4.4 Voeding
De geïncludeerde zwangere vrouwen mogen zowel borstvoeding of flesvoeding geven. Probiotica of placebo worden toegevoegd aan het normale eetpatroon van de proefpersoon. Er zijn m.a.w. geen restricties wat betreft dieet behalve dat de proefpersonen géén gebruik mogen maken van probiotica-bevattende producten.

4.5 Methoden en uitkomstvariabelen
<u>Probiotica</u>: probiotica (keuze wordt gemaakt op basis van de resultaten van in-vitro studie en dierstudie) worden gegeven aan de moeders vanaf 4-6 weken antepartum en aan hun hoog-risico pasgeborenen gedurende het eerste levensjaar in een dagelijkse dosis van 3×10^9 CFU. Hetzelfde geld voor het placebo preparaat.

<u>Uitkomstvariabelen (gebruikte referenties zie 8c):</u>
De uitkomstvariabelen en de tijdstippen van meting zullen per vraagstelling worden beschreven en toegelicht.

4.5.1 <u>Hoofdvraagstelling A</u>: Wat is het effect van vroege toediening van probiotica op allergische aandoeningen op de kinderleeftijd?

De primaire uitkomstvariabele ten aanzien van deze vraag is de aanwezigheid van allergische aandoeningen op de leeftijd van 2 jaar. Deze uitkomstvariabele wordt bepaald op de leeftijd van 3, 12 en 24 manden (zie tijdschema). Allergische aandoeningen zijn als volgt gedefinieerd: [9]

- Eczeem: diagnose van eczeem of atopische dermatitis is gebaseerd op de Sampson criteria: erytheem, oedeem, krabeffecten (excoriaties) en jeuk gedurende tenminste 4 weken.[10] De ernst wordt bepaald met behulp van de Scoring Atopic Dermatitis (SCORAD).[11]
- Voedselallergie: Voedselallergie wordt gedefinieerd als klinische manifestaties van erythemateuze, papulaire, macrovesiculaire huiduitslag, diarree, braken of ademhalingsproblemen in relatie tot inname van bepaald voedsel. Voor de definitieve diagnose is een positieve huidpriktest of verhoogd specifiek IgE en een eliminatie-provocatie-reëliminatie procedure noodzakelijk.
- Astma: astma is moeilijk te definiëren bij kinderen op de leeftijd van 2 jaar. Bij kinderen onder de 3 jaar worden 3 verschillende fenotypen van bronchusobstructieve aandoeningen beschreven.[12] Recidiverende klachten van bronchusobstructie in de aanwezigheid van atopie (gedefinieerd als verhoogd totaal of specifiek IgE) ontwikkelen zich meest waarschijnlijk later tot chronisch astma. Daarom zal de aanwezigheid van atopie voor of op de leeftijd van 2 jaar in combinatie met terugkerende klachten van bronchusobstructie worden aangeduid als astma. Veranderingen in de ernst van astma worden bepaald aan het gebruik van kortwerkende beta-2-agonisten. Aangezien op de leeftijd van 4-5 jaar een meer definitieve diagnose van astma kan worden gesteld zal op deze leeftijd de follow up van het cohort kinderen worden uitgevoerd.
- Rhinitis: rhinitis wordt gedefinieerd als terugkerende perioden van waterige rhinorrhoe (neusvloed) in combinatie met frequent niezen, jeuk aan de neus, roodheid van de ogen en tranende ogen. De diagnose wordt bevestigd met de aanwezigheid van verhoogd specifiek IgE. Veranderingen in de ernst worden afgemeten aan subjectieve beleving en gebruik van medicijnen die de klachten verminderen.

Symptoomscore

Ouders wordt gevraagd een dagboek bij te houden met daarin gegevens over symptomen van allergische aandoeningen, infectieuze ziekten (inclusief gastroenteritis), bezoek aan de huisarts (inclusief diagnose), voorgeschreven medicijnen en gebruik daarvan, veranderingen in voedselpatroon tot de leeftijd van 2 jaar. Bij de long-term follow up zal contact worden opgenomen met de behandelende huisartsen ter evaluatie van doktersbezoek en medicijngebruik naar aanleiding van allergische en/of astmatische klachten. Elk gepland bezoek aan de polikliniek zal bestaan uit een uitgebreide anamnese naar de symptomen van allergische aandoeningen. Veranderingen in de ernst van symptomen worden bepaald aan het gebruik van medicijnen en de hoeveelheid daarvan.

Algemeen Lichamelijk onderzoek

Tijdens elk bezoek aan de polikliniek wordt een algemeen lichamelijk onderzoek verricht, met nadruk op symptomen van allergische aandoeningen. Aanwezigheid of veranderingen van eczeem worden bepaald met gebruik van scoringsmethoden (SCORAD).

Huidpriktesten

Als aanvullend, meer sensitief, onderzoek naar het voorkomen van allergie, zullen op de leeftijd van 2 jaar bij alle deelnemende kinderen huidpriktesten worden uitgevoerd in samenwerking met de polikliniek van het Centrum voor Kinderallergologie.

Afgeleide vraagstellingen (secundaire uitkomstvariabelen)

4.5.2 B: Leidt vroege toediening van probiotica tot een blijvende andere samenstelling van de darmflora ten opzichte van de placebogroep?

Om deze vraag te beantwoorden zal plaatsvinden:

1. Afname oppervlaktekweken en verzamelen uitgezogen sputum postpartum.
2. Vanaf dag 2 post partum 1x/week gedurende 4 weken afname faeces portie.
3. Na staken probiotica/placebo op leeftijd 12 maanden 1x/week gedurende 4 weken afname faecesportie.
4. Afname 1 faecesportie op de leeftijd van 3, 18 en 24 maanden

Ad 1: het kind wordt tijdens de geboorte gekoloniseerd door de flora van de moeder. Door middel van oppervlakte kweken postpartum wordt deze kolonisatie bepaald en gekeken of er verschil is tussen probiotica groep en placebo groep.

Ad 2 t/m 4: om de kolonisatie van de darmflora te volgen is gekozen voor frequente faeces monsters, met name rondom de start van toediening van probiotica/placebo aan het kind en het staken daarvan op de leeftijd van 12 maanden.

De humane intestinale flora wordt gedomineerd door anaërobe micro-organismen, die lastig en tijdrovend zijn om te kweken, met als gevolg dat vele stammen vooralsnog niet kunnen worden gekweekt. Er zal daarom in deze studie gebruik gemaakt worden van moleculaire methoden en technieken, gebaseerd op 16S rRNA genen, die onafhankelijk zijn van kweken en waarmee het mogelijk is tijdens de studie veranderingen van de intestinale flora te volgen.

PCR en "denaturing gradient gel electrophoresis (DGGE) van 16S rDNA genen
(Favier CF, Vaughan EE et al. Molecular Monitoring of Succession of Bacterial Communities in human neonates. Appl Environ Microbiol 2002;68:219-6)
Dit is een kwalitatieve analyse waarmee de dominante species in de faeces kunnen worden aangetoond en worden vervolgd in de tijd. Faeces monsters worden door de ouders thuis verzameld, ingestuurd naar het WKZ en daar ingevroren. DNA wordt geïsoleerd uit de faeces monsters. Bacterieel 16S DNA wordt geamplificeerd met PCR en de PCR fragmenten worden gescheiden met DGGE. De resulterende profielen worden gebruikt om veranderingen van de flora te vervolgen gedurende de tijd. Binnen deze techniek kan er gekeken worden naar specifieke populaties, *Bifidobacterium*, *Lactobacillus* species, met genus specifieke PCR en DGGE. Dit betekent dat de ingenomen probiotische stam kan worden vervolgd.
Fluorescentie in situ hybridisatie (FISH) in combinatie met flowcytometrie
(Vaughan EE et al. The intestinal LABs. 2002 Antonie van Leeuwenhoek 82:341-352)
Bacteriën in de faeces worden gehybridiseerd met fluorescent gelabelde 16S rRNA probes specifiek voor een type bacterie. Op dit moment zijn er 6 gevalideerde probes beschikbaar , waaronder voor bifidobacteria, lactobacilli, clostridium en bacteroides. Met flowcytometrie kunnen de gelabelde populaties onderscheiden worden van de ongelabelde populaties en kan een quantitatieve analyse worden gedaan naar de samenstelling van de darmflora.

4.5.3 *C: Geeft vroege toediening van probiotica in-vivo modulatie van het immuunsysteem? Vindt er onderdrukking van plaats van Thelper-2-cel responsen of worden regulatoire T cellen geïnduceerd?*

Van de pasgeborenen worden de parameters op tijdstip 0 (=geboorte) bepaald in navelstrengbloed en beschouwd worden als baseline parameters. Daarna wordt tijdens de follow-up bezoeken van 3, 12, 24 maanden en 5 jaar perifeer bloed afgenomen bij de kinderen. De parameters waarnaar gekeken zal worden zijn totaal IgE en specifiek IgE, morfologie mononucleaire cellen (inclusief toll-like receptoren), bepaling van productie van cytokinen. Deze uitkomstvariabelen worden hieronder afzonderlijk beschreven.

IgE

Totaal en specifiek IgE wordt bepaald door middel van immunoCAP techniek (Pharmacia, Nederland). Specifiek IgE tegen de 6 belangrijkste allergenen (koemelkeiwit, huisstofmijt, kat, hond, gras- en boompollen) zullen worden bepaald.

Morfologie mononucleaire cellen

Voor lymfocyten fenotypering wordt gebruik gemaakt van CD3, CD4, CD8, CD45RA, CD45RO. Om onderscheid te maken tussen Th1 en Th2 wordt gekeken naar expressie van CD30, CCR3, CCR4, CCR5 en CCR8. Regulatoire T cellen worden onderscheiden met de markers CD4, CD25, CTLA-4, GITR, L-selectin, CCR4, CD45RB en CD45RO.
Expressie van toll-like receptoren (TLR) op mononucleaire cellen uit perifeer bloed worden bepaald met flow cytometrie met gebruik van antilichamen tegen TLR (commercieel verkrijgbaar: TLR2, -3, -4, -6, -9).

Cytokinen bepalingen

Volbloed wordt verdund met RPMI medium (GIBCO/BRL). Cellen worden gestimuleerd met LPS en IFN-gamma. Na 18 uur worden de supernatanten afgenomen en ingevroren ter bepaling van cytokinen met Luminex technologie. Cellen worden daarnaast gestimuleerd met medium (RPMI), CD2/CD28 en PHA. Na 48 en 72 uur worden de supernatanten afgenomen en ingevroren. Proliferatie van de cellen worden op 48 en 72 uur bepaald met incorperatie van 3H-thymidine. Cytokinen worden bepaald in de supernatanten met Luminex (*De Jager W et al. Simultaneous detection of 15 human cytokines in a single sample of stimulated peripheral blood mononuclear cells. Clin Diagn Lab Immunol 2003;10:133-9)*. Met deze techniek kunnen in 50 microliter supernatant 15 verschillende cytokinen en 5 verschillende chemokinen bepaald worden. De sensitiviteit van deze techniek is vergelijkbaar met een conventionele ELISA, intra-assay CV <10% en interassay CV <20%.

4.5.4 D: Spelen genetische polymorfismen van moleculen en receptoren van het immuunsysteem een bepalende rol in de respons op probiotica ("gene by environment interaction")?

Polymorfismen van cytokine genen en andere genen betrokken bij de innate en adaptive immuniteit worden onderzocht (*Lazarus R et al. Single nucleotide polymorfisms in innate immunity genes: abundant variation and potent role in complex human disease. Immunological Reviews 2002;190:9-25)* . Daartoe zal in een samenwerkingsverband met Dr. Ruven en Grutters (Antonius Ziekenhuis, Nieuwegein) dmv sequentie specifieke primer PCR, polymorfismes van IL-4, IL-4R, IL-6, Il-10, Il-10R, Il-13, Il1-β, TNFα, TNFRI, TNFR2, INFγ, INFγR1, INFγR2, CC16, CD14, Toll-like receptor 4, NRAMP, MBL, FcγRIIa, FcγRIIIa and IIIb worden bepaald. Met elk van deze genen is binnen het UMC specifieke ervaring. Uiteindelijk zal de klinische uitkomst van de primaire preventie worden gecorreleerd aan polymorfisme profielen.

5. Probiotica preparaat

Probiotica zijn “voedingssupplementen op basis van levende micro-organismen (met name melkzuurbacteriën) met een “gezondheidsbevorderend” effect voor de gastheer. Van probiotica is bekend dat ze (over-)groei van bepaalde pathogenen kunnen remmen en de barrière functie van de darm kunnen verbeteren. Bovendien is bekend dat bepaalde probiotica ons immuunsysteem op een positieven manier kunnen beïnvloeden.

Ontwikkeling

Er is in deze studie een multispecies probiotica preparaat ontworpen in samenwerking met Winclove Bio Industries B.V. ® te Amsterdam. Het preparaat bestaat uit 3 bekende en in zuivelproducten voorkomende probiotische stammen die geselecteerd zijn uit een stammenbank van ruim 75 probiotische stammen. Van al deze stammen zijn individuele eigenschappen bekend zoals stabiliteit in het eindproduct, overleving in het maagdarmkanaal en antibiotica resistentie. Op basis van de individuele eigenschappen zijn uit deze 75 stammen 14 stammen geselecteerd om verder te karakteriseren (zie hoofdstuk 6). Er is gekozen voor een multispecies preparaat in plaats van een preparaat bestaande uit 1 stam omdat een multispecies preparaat het voordeel kan hebben van synergie of symbiose tussen de verschillende stammen waardoor het effect van het totale preparaat kan worden versterkt.

Selectiecriteria

Op basis van het vooronderzoek (zie hoofdstuk 6) zijn 3 stammen geselecteerd om als multispecies probiotica preparaat te gaan gebruiken in de onderhavige studie. Kort samengevat zijn de stammen geselecteerd op basis van de hierboven genoemde individuele eigenschappen, in-vitro capaciteit om anti-inflammatoire cytokinen (IL-10) te induceren en produktie van allergie gecorreleerde cytokinen te onderdrukken (IL-4, IL-5 en IL-13), hun capaciteit om te hechten aan dendritische cellen en vanuit het oogpunt van veiligheid en hun aanwezigheid in reeds bestaande zuivelproducten.

Geselecteerde stammen

1. *Bifidobacterium bifidum*
2. *Bifidobacterium Infantis*
3. *Lactococcus lactis*

Toediening

Het probiotica product zal 1 maal daags worden toegediend in een totale dosis van 3 x 10^9 Colony Forming Units (CFU). Voor een preparaat bestaande uit 3 stammen betekent dit 1 x 10^9 CFUs per stam. De zuigelingen krijgen borstvoeding of flesvoeding (afhankelijk van de keuze van de ouders). Het probiotica preparaat wordt toegediend in flesjes van 10 ml met daarin de voeding van keuze van de ouders. Deze wordt gegeven voor de borstvoeding of flesvoeding. De drager van de probiotica stammen zal bestaan uit rijstzetmeel aangezien die hypo-allergeen is. De drager zal bestraald worden met gamma-stralen om contaminatie van natuurlijk aanwezige bacteriën te voorkomen.

Placebo

Het placebo preparaat bestaat uit de voedingsmatrix, dus hydrolysaat melk en de drager van het probioticum bestaande uit rijstzetmeel.

Veiligheid

Er wordt uitsluitend gebruik gemaakt van probiotische bacterie stammen die sinds langere tijd reeds gebruikt worden in de voedingsindustrie voor de produktie van voedingssupplementen en zuivelproducten als kaas, kwark en yoghurt. De meeste probiotische stammen worden gezien als commensale microorganismen zonder pathogene werking. In recente artikelen worden de veiligheid van *Lactobacilli* en *Bifidobacteria* beschreven [36, 37]. Deze artikelen geven een review van de beschikbare literatuur en beschrijven dat *Lactobacilli* en *Bifidobacteria* in zeldzame gevallen de oorzaak zijn van infectie bij zowel gezonde als immuungecompromitteerde individuen. Een uitgebreide literatuurstudie naar infectieuze complicatie van specifieke probiotische stammen leverde publicaties op over 2 probiotische stammen, *Lactobacillus rhamnosus* en *Enterococcus faecium*. Alhoewel het onduidelijk is of deze infecties veroorzaakt werden door de probiotische stam of een gelijknamige commensale stam, zullen deze stammen niet worden gebruikt.
Op basis van de ervaringen van toediening van probiotica bij kinderen, met name zijn studies verricht van toediening van probiotica bij infectieuze diarree, worden geen ongewenste bijwerkingen of serious adverse events verwacht. We hebben voor deze studie een serious adverse avents formulier samengesteld waarvan bij het voorkomen van ongewenste bijwerkingen en serious adverse avents dit beoordeeld zal worden door de hoofdonderzoeker (Dr. M.O. Hoekstra) en direct gemeld worden aan de METC door de hoofdonderzoeker.

6. Vooronderzoek

In vitro studie

"In-vitro effects of probiotics on proliferation, morphology and cytokine production on cord-blood derived mononuclear cells from high-risk newborns". (Drs. L.E.M. Niers, Dr. G.T. Rijkers, Dr. M.O. Hoekstra, Algemene Pediatrie, WKZ, UMC Utrecht)

Op basis van deze studie zijn de probiotische stammen met de meest gunstige effecten op het immuunsysteem ten aanzien van preventie en onderdrukking van allergie (lees: type-2-Thelper cel responsen) geselecteerd en worden gebruikt in het beschreven onderzoeksvoorstel. Door onze expertise op het gebied van immunoregulatie bij kinderen en multiplex cytokinen onderzoek hebben we op dit onderzoeksterrein contacten met Numico (Dr. R. van Tol) en Winclove (Dr. P. Pekelharing), belangrijke industriële producenten van probiotica.

In deze studie zijn op basis van de individuele eigenschappen (als stabiliteit in het eindproduct, overleving in het maagdarmkanaal en antibiotica resistentie) van de 75 stammen van Winclove Bioindustries, Amsterdam, 14 stammen geselecteerd om verder te karakteriseren. De 14 geteste probiotica hebben stamspecifieke eigenschappen voor wat betreft invloed op mononucleaire cellen afgemeten aan productie van cytokinen. De 3 stammen die gebruikt worden in de onderhavige studie, zijn geselecteerd op basis van hun vermogen om de productie van anti-inflammatoire cytokinenen (IL-10) te induceren, de productie van astma en allergie gerelateerde cytokinen (IL-4, IL-5, IL-13) te onderdrukken en minder produktie van pro-inflammatoire cytokinen induceren ten opzichte van de overige 11 stammen. Tevens is de capaciteit van de 14 stammen om aan dendritische cellen te binden onderzocht. Hierbij zijn we uitgegaan van onze hypothese dat het contact tussen de "gastheer" en probiotische stammen cruciaal is voor een gunstige werking van deze bacteriën. Dit contact wordt tot stand gebracht in de darm waarbij de link tussen de bacterie in het darmlumen of op de darmwand en het immuunsysteem van de gastheer gevormd wordt door dendritische cellen, die aanwezig zijn in de wand van de darm. De drie geselecteerde stammen kunnen in-vitro goed binden aan dendritische cellen.

Met de keuze van de stammen is vanuit het oogpunt van veiligheid rekening gehouden met de aanwezigheid van deze stammen in reeds op de markt zijnde voedingssupplementen en zuivelproducten. Voor de 3 geselecteerde stammen geldt bijvoorbeeld dat ze aanwezig zijn in het voedingssupplement orthiflor en dat 1 van de stammen aanwezig is in het zuivelproduct Mona Vifit.

7. Onderzoekslijnen UMCU waar PANDA studie op aansluit

Het beschreven onderzoek maakt deel uit van een onderzoekslijn binnen het Wilhelmina Kinderziekenhuis naar de mogelijkheden van het gebruik van probiotica bij kinderen. Binnen het UMC Utrecht zijn de verschillende onderzoeken naar de mogelijkheden van probiotica verenigd in het Probiotica Platform Utrecht, wat bestaat uit een samenwerkingsverband tussen de divisies Algemene Kindergeneeskunde, Laboratorium Pediatrische Immunologie, Afdeling Dermato/ Allergologie, Heelkunde en de Faculteit Farmacie. Naast dit onderzoeksvoorstel omvat het Probiotica Platform Utrecht de volgende studies:

- *In vitro studie* "In-vitro effects of probiotics on proliferation, morphology and cytokine production on cord-blood derived mononuclear cells from high-risk newborns". (Drs. L.E.M. Niers, Dr. G.T. Rijkers, Dr. M.O. Hoekstra, Algemene Pediatrie, WKZ, UMC Utrecht)
  METC protocol nummer: 03/035

- *Diermodel*: "The effect of different (combinations) of probiotics on several immune parameters in an ovalbumen-astma mouse model" (Drs. C. Severijnen, Dr. A. van Oosterhout, Dr. M.A. Bloksma en Dr. M.O. Hoekstra, Afdeling Farmacologie, UMC Utrecht)

- *Tertiaire preventie pilotstudie* met probiotica bij kinderen met constitutioneel eczeem (Dr. E. van Hoffen, Dermatologie/Allergologie, AZU, UMC Utrecht).
  METC protocol nummer: 02/162

- *Preventie van bovenste luchtweginfecties* door probiotica en prebiotica bij kinderen die kinderdagverblijf bezoeken (Dr. E.A.M. Sanders, Pediatrische Immunologie en Reumatologie WKZ, UMC Utrecht).

- *Multicentre trial* naar de effecten van probiotica bij pancreatitis (PROPATRIA studie) en studie naar effecten van probiotica in een pancreatitis rattenmodel (Prof. Dr. L.M.A. Akkermans en Prof. Dr. H.G. Gooszen, afdeling Heelkunde en Gastrointestinale Research Afdeling, AZU, UMC Utrecht). Start september 2003.
  METC protocol nummer: 03/169

8. Ethische overwegingen

De studie zal uitgevoerd worden in overeenstemming met de ethische principes van de Verklaring van Helsinki en na de goedkeuring van de onderhavige studie door de Medisch Ethische Toetsingscommissie van het UMC Utrecht. Het UMC Utrecht heeft, als verrichter (opdrachtgever) van dit onderzoek, een risicoverzekering afgesloten voor proefpersonen die meedoen aan wetenschappelijk onderzoek. Informatie over deze verzekering wordt bijgevoegd bij de patiënten informatiebrief en is als bijlage IV te vinden in dit onderzoeksprotocol.

Informed consent
De zwangere vrouw met haar aanstaande zoon / dochter die aan de inclusiecriteria voldoen en interesse heeft in deelname aan de studie, zullen om toestemming gevraagd worden voor deelname aan dit onderzoek. Voor de pasgeborene als wilsonbekwame persoon betekent dit dat de ouder(s) of wettelijk vertegenwoordiger hiervoor toestemming zal moeten verlenen. De opzet van de studie zal door de onderzoekster en door middel van de patiënten informatiebrief worden uitgelegd. De zwangere vrouw verleent door middel van ondertekening van een informed consent formulier medewerking aan dit onderzoek. De (aanstaande) ouders dan wel wettelijk vertegenwoordiger van de aanstaande pasgeborene geven door middel van ondertekening van het daartoe behorende informed consent formulier hun toestemming voor deelname van hun kind aan dit onderzoek. Hierbij houden de deelnemers de mogelijkheid zich te allen tijde uit het onderzoek terug te trekken, zonder dat de zorg voor hen daardoor veranderd. De gegevens van de deelnemers zullen centraal verzameld worden onder vermelding van een studienummer. Slechts de onderzoekscoördinatoren (Dr. M.O. Hoekstra en Dr. G.T. Rijkers) beschikken over een lijst met namen. Aldus is de privacy van de deelnemers gewaarborgd.

Belasting voor de deelnemers
De belasting voor de deelnemers zal voornamelijk gelden voor de pasgeborenen. Zij worden gedurende de follow-up tot de leeftijd van 2 jaar 3 maal geprikt voor een bloedafname. Tevens zal op de leeftijd van 2 jaar met huidpriktesten aanvullend onderzoek gedaan worden naar het voorkomen van sensibilisatie bij de kinderen. De onderzoeksgroep realiseert zich terdege de belasting die vena puncties betekenen voor kinderen en voor de ouders. De belasting wordt zoveel mogelijk beperkt door de venapunctie uit te laten voeren door iemand met ervaring in bloedprikken bij kinderen en door de plaats van prikken van tevoren te verdoven met emla zalf. Ten aanzien van huidpriktesten realiseert de onderzoeksgroep zich ook terdege dat die als belastend kunnen worden ervaren door de kinderen en ouders. Onze ervaring leert dat de huidpriktesten door kinderen in de betreffende leeftijdscategorie over het algemeen niet als belastend of oncomfortabel wordt ervaren, in tegenstelling wat op basis van de handelswijze en uitvoering van dit allergie onderzoek verwacht zou kunnen worden. Indien de zuigeling zich dusdanig verzet of oncomfortabel is en de ouder(s) / wettelijk vertegenwoordiger verzoekt om het prikken of ander aanvullend onderzoek te staken, dan zal dit verzoek vanzelfsprekend worden gehonoreerd. De belasting voor de ouders wordt als minimaal ingeschat. Het betreft voornamelijk het verzamelen van ontlasting en het bijhouden van klachten bij hun deelnemende kind.

Probiotica

Probiotica worden over het algemeen goed verdragen. In een enkel geval kan een onprettig gevoel ontstaan door overmatige productie van gassen. Mochten er bijwerkingen optreden dan zal hiervan melding worden gemaakt aan de METC. Indien nodig zal de toedieningen in overleg met de deelnemers gestaakt worden.

9. Organisatie

In onderstaande tabel zijn de betrokken personen bij dit onderzoek aangegeven. Het onderzoek wordt uitgevoerd door Mw. L.E.M. Niers, arts-assistent Kindergeneeskunde. Het onderzoek zal begeleid worden door Dr. M.O. Hoekstra en Dr. G.T. Rijkers. De immunologische uitkomstvariabelen zullen bepaald worden in het Laboratorium voor Pediatrische Immunologie, in samenwerking met Mw. N.O.P van Uden en Dr. G. van Bleek. Aangezien het onderzoek onderdeel uitmaakt van een samenwerkingsverband tussen de Divisie Kindergeneeskunde en de afdeling Dermatologie/Allergologie van het AZU is Dr. E.F. Knol betrokken bij het onderzoek.
Voor het onderzoek in de ontlasting wordt gebruik gemaakt van de expertise van de Universiteit van Wageningen, Mw. Dr. E.E. Vaughan.
Voor de statistische analyse van de uitkomstvariabelen is Dr. C.S.P.M. Uiterwaal betrokken.
Prof. M.L Kapsenberg en Mw. Dr. H.H. Smits hebben een grote expertise op het gebied van probiotica en antigeen presentatie en hebben een adviserende rol in het onderzoek

Naam (met titel)	discipline	t.l.v. inst/vakgr.	uren/week
Mw. Drs. L.E.M. Niers	Arts	UMCU	40
Dr. M.O. Hoekstra	Kinderarts	UMCU	6
Prof. J.L.L. Kimpen	Kinderarts en opleider	UMCU	2
Prof. W. Kuis	Kinderarts, immunoloog	UMCU	adv
Dr. G.T. Rijkers	Immunoloog, Hoofd Lab. Pediatrische Immunologie	UMCU	6
Dr. G. van Bleek	Immunoloog	UMCU	2
Mw. Drs. N.O.P van Uden	Research analist (WO)	UMCU	20
Dr. E.F. Knol	Immunoloog, Hoofd onder-zoek Dermato/Allergologie	UMCU	2
Mw. Dr. E.E. Vaughan	Microbioloog, Lab. Microbiologie Wageningen	WUR	1
Dr. C.S.P.M. Uiterwaal	Statisticus/epidemioloog	UMCU	adv
Prof. M.L. Kapsenberg	Immunoloog	AMC	adv
Mw. Drs. H.H. Smits	Med. Bioloog/Immunoloog	AMC	adv

10. Literatuur

Hygiene hypothesis

1. Umetsu DT, McIntire JJ, Akbari O et al. Asthma: an epidemic of dysregulated immunity. Nat Immunol 2002;3:715-20.
2. Von Mutius E. Pro: the increase in asthma can be ascribed to cleanliness. Am J Respir Crit Care Med 2001:164:1106-7.
3. Platts-Mills TA, Woodfolk JA, Sporik RB. Con: the increase in asthma cannot be ascribed to cleanliness. Am J Respir Crit Care Med 2001;164:1107-8.
4. Weiss ST. Eat dirt: the hygiene hypothesis and allergic disease. N Eng J Med 2002;347:930-1.
5. Wills-Karp M, Santeliz J, Karp CL. The germless theory of allergic disease: revisiting the hygiene hypothesis. Nat Rev Immunol 2001;1:69-75.

Timing: sensitization in utero and early life

6. Bjorksten B. Allergy priming early in life. Lancet. 1999;353:167-8.
7. Warner JO, Jones CA, Kilburn SA et al. Prenatal sensitization in humans. Pediatr Allergy Immunol 2001;11S13:6-8.
8. Herz U, Joachim R, Ahrens B et al. Allergic sensitization and allergen exposure during pregnancy favor the development of atopy in the neonate. Int Arch Allergy Clin Immunol 2001;124:193-6.

Allergic disease in childhood

9. Laan MP et al. Markers for early sensitization and inflammation in relation to clinical manifestations of atopic disease up to 2 years of age in 133 high-risk children. Clin Exp Allergy 2000;30:944-53.
10. Sampson HA. Pathogenesis of eczema. Clin Exp Allergy 1990;20:459-67.
11. Sprikkelman AB et al. Severity scoring in allergic dermatitis:a comparison of three scoring systems. Allergy 1997;52:44-9.
12. Martinez FD. Development of wheezing disorders and asthma in preschool children. Pediatrics 2002;109(S2):362-7.

Role of the intestinal flora in the development of allergy and atopy

13. Bjorksten B, Naaber P, Seppe E et al. The intestinal microflora in allergic Estonian and Swedish 2-year-old children. Clin Exp Allergy. 1999;29:342-6.
14. Kalliomaki M, Kirjavainen P, Eerola E et al. Distinct patterns of neonatal gut microflora in infants in whom atopy was and was not developing. J Allergy Clin Immunol. 2001;107:129-34.
15. Kalliomaki M, Isolauri E. Role of intestinal flora in the development of allergy. Curr Opin Allergy Clin Immunol 2003;36:223-7.

Reviews or editorials addressing the use of probiotics

16. Mursh SH. Toll of allergy reduced by probiotics. Lancet 2001;357:1057-9.
17. Isolauri E, Sutas Y, Kankaanpaa P et al. Probiotics: effects on immunity. Am J Clin Nutr. 2001;73:44S-50S
18. Erickson KL, Hubbard NE. Probiotic immunomodulation in health and disease. J Nutr. 2000;130:403S-9S
19. Matricardi PM. Probiotics against allergy: data, doubts and perspective. Allergy 2002;57:185-7.

In-vitro effects of probiotics

20. Hessle C, Hanson LA, Wold AE. Lactobacilli from human gastrointestinal mucosa are strong stimulators of IL-12 production. Clin Exp Immunol. 1999;116:276-82.
21. Christensen HR, Frokiaer H, Pestka JJ. Lactobacilli differentially modulate expression of cytokines and maturation surface markers in murine dendritic cells. J Immunol. 2002;168:171-8.
22. Miettinen M, Vuopio-Varkila J, et al. Production of human tumor necrosis factor alpha, interleukin-6 (IL-6), and IL-10 is induced by lactic acid bacteria. Infect Immun.1996;64:5403-5.
23. Miettinen M, Matikainen S, Vuopio-Varkila J et al. Lactobacilli and streptococci induce interleukin-12 (IL-12), IL-18 and gamma interferon production in human peripheral blood mononuclear cells. Infect Immun 1998;66:6058-62.
24. Pessi T, Sutas Y, Hurme M et al. Interleukin-10 generation in atopic children following oral Lactobacillus Rhamnosus GG. Clin Exp Allergy 2000;30:1804-8.
25. Pochard P, Gosset P, Grangette C et al. Lactic acid bacteria inhibit Th2 cytokine production by mononuclear cells from allergic patients. J Allergy Clin Immunol 2002;110:617-23.

Clinical trials probiotics in allergic disease
26. Kalliomaki M, Salminen S, Poussa T et al. Probiotics and prevention of atopic disease: 4 year follow-up of a randomised placebo-controlled trial. Lancet 2003;361:1869-71. (*primary prevention*)
27. Kalliomaki M, Salminen S, Arvilommi H et al. Probiotics in primary prevention of atopic disease: a randomised placebo-controlled trial. Lancet 2001;357:1076-79. (*primary prevention*)
28. Kirjavainen PV, Salminen SJ, Isolauri E. Probiotic bacteria in the management of atopic disease: underscoring the importance of viability. J Pediatr Gastroenterol Nutr 2003;36:223-7.
29. Majamaa H, Isolauri E. Probiotics: a novel approach in the management of food allergy. J Allergy Clin Immunol. 1997;99:179-85.
30. Isolauri E, Arvola T, Sutas Y et al. Probiotics in the management of atopic eczema. Clin Exp Allergy. 2000;30:1604-10.
31. Kalliomaki M, Ouwehand A, Arvilommi H et al. Transforming growth factor-beta in breast milk: a potential regulator of atopic disease at an early age. J Allergy Clin Immunol. 1999:104:1251-7.
32. Rautava S, Kalliomaki M, Isolauri E. Probiotics during pregnancy and breastfeeding might confer immunomodulatory protection against atopic disease in the infant. J Allergy Clin Immunol 2002;109:119-21.
33. Rosenfeldt V, Benfeldt E, Nielsen SD et al. Effect of probiotic Lactobacillus strains in children with atopic dermatitis. J Allergy Clin Immunol 2003;111:389-95.
34. Helin T, Haahtela S, Haahtela T. No effect of oral treatment with an intestinal bacterial strain, Lactobacillus rhamnosus (ATCC 53103), on birch-pollen allergy: a placebo-controlled double-blind study. Allergy. 2002;57:243-6.

Gene by environment interaction
35. Smit JJ, Loveren H van, Hoekstra MO et al. Influence of the macrophage bacterial resistance gene, Nramp1 (Slc11a1), on the induction of allergic asthma in the mouse. FASEB J 2003 17:958-60.

Safety of probiotics
36. Borriello SP, Hammes WP, Holzapfel W et al. Safety of probiotics that contain lactobacilli and bifidobacteria. Clin Infect Dis. 2003;36:775-80.
37. Sipsas NV, Zonios DI, Kordossis T. Safety of Lactobacillus strains used as probiotic agents. Clin Infect Dis 2002; 34: 1283-1284

Bijlage I

VRAGENLIJST VOOR DEELNEMERS AAN DE PANDA STUDIE

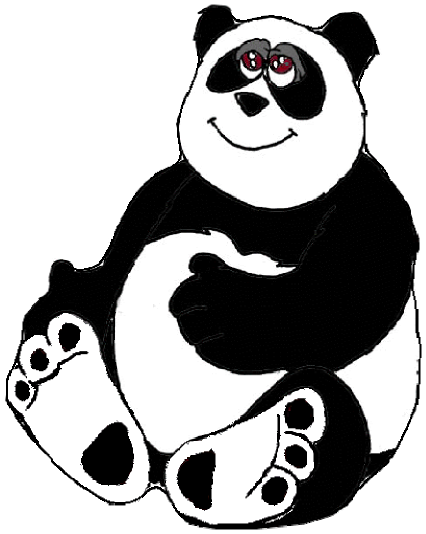

Probiotics AND Allergy

Toelichting bij de vragenlijst

De vragenlijst is ontwikkeld om een beeld te krijgen van het voorkomen van voedselallergie, hooikoorts, allergische rhinitis, astma en andere allergische klachten bij deelnemers of gezinnen van de deelnemers aan de PANDA studie

Bij de vragen hoeft u alleen het juiste antwoord te kiezen en het betreffende hokje aan te kruisen. Indien u het lastig vindt om een keuze te maken tussen 'ja' of 'nee' is het voor het onderzoek beter als u in zo'n geval toch een keuze maakt en het antwoord aankruist dat het beste van toepassing is. U kunt uw twijfel kort toelichten bij de desbetreffende vraag.

Met het oog op privacy worden de gegevens zonder uw naam en adres in de computer verwerkt en alleen onder uw deelnemersnummer ingevoerd.

Voor nadere informatie kunt u bellen met:

Titia Niers, arts-assistent Kindergeneeskunde, Wilhelmina Kinderziekenhuis Utrecht
030-2504000, sein 5063 OF 0302504582

ALGEMENE GEGEVENS DEELNEMERS PANDA STUDIE
BIJ INCLUSIE

(in te vullen door onderzoekster)

Naam moeder:

Geboorte datum moeder:

Geboorte datum vader:

Geboorte datum / data kinderen: -
-
-
-

Datum laatste menstruatie moeder:

A terme datum moeder:

Gravida: /Para:

STUDIE NUMMER Deelnemer (in te vullen door onderzoekster):

VRAGENLIJST ASTMA EN ALLERGISCHE AANDOENINGEN

Vraag 1

A. Bent u bij de huisarts bekend met astma en / of allergie? **JA [ ] NEE [ ]**
B. Is uw echtgenoot / partner bij de huisarts bekend met astma en / of allergie **JA [ ] NEE [ ]**
C. Heeft u een kind, die bekend is bij de huisarts met astma en / of allergie **JA [ ] NEE [ ]**

Vraag 2

A. Zijn er bij u ooit allergietesten verricht, door middel van bloedonderzoek of testen op de huid? **JA [ ] NEE [ ]**
B. Zijn er bij uw echtgenoot / partner ooit allergietesten verricht, door middel van bloedonderzoek of testen op de huid? **JA [ ] NEE [ ]**
C. Zijn er bij één of meerdere ouder(e) kind(eren) ooit allergietesten verricht, door middel van bloedonderzoek of testen op de huid? **JA [ ] NEE [ ]**

Vraag 3

A. Gebruikt u nu of heeft u in het verleden medicatie voor astma gebruikt, de zogenaamde pufjes? **Zo ja**, kunt u aangeven welke medicijnen en in welke dosering?…………………………………………………………………
……………………………………………………………………………………
…………………………………………………………………………………… **JA [ ] NEE [ ]**
B. Gebruikt uw echtgenoot / partner nu of heeft hij in het verleden medicatie voor astma gebruikt, de zogenaamde pufjes? **Zo ja**, kunt u aangeven welke medicijnen en in welke dosering?………………………………………………
……………………………………………………………………………………
…………………………………………………………………………………… **JA [ ] NEE [ ]**

C. Gebruikt één of meerdere ouder(e) kind(eren) nu of in het verleden medicatie voor astma gebruikt, de zogenaamde pufjes? **Zo ja**, kunt u aangeven welke medicijnen en in welke dosering?………………………………………………
……………………………………………………………………………………
…………………………………………………………………………………… **JA [ ] NEE [ ]**

Vraag 4

A. Gebruikt u of heeft u medicatie gebruikt om klachten van allergie te verminderen, bijvoorbeeld een anti-histaminicum? Zo ja, kunt u aangeven welke?
……………………………………………………………………………………… **JA [ ] NEE [ ]**
B. Gebruikt uw echtgenoot / partner of heeft hij medicatie gebruikt om klachten van allergie te verminderen? Zo ja, welke?………………………………
……………………………………………………………………………………… **JA [ ] NEE [ ]**
C. Gebruikt één of meerdere ouder(e) kind(eren) ooit medicatie om klachten van allergie te verminderen? Zo ja, welke?………………………………
………………………………………………………………………………… **JA [ ] NEE [ ]**

Vraag 5

A. Heeft u last van hooikoorts? **JA [ ] NEE [ ]**
B. Heeft uw echtgenoot / partner last van hooikoorts **JA [ ] NEE [ ]**
C. Heeft één of meerdere ouder(e) kind(eren) last van hooikoorts **JA [ ] NEE [ ]**

Vraag 6

A.	Heeft u ooit eczeem gehad?	**JA [ ] NEE [ ]**
B.	Heeft uw echtgenoot / partner ooit eczeem gehad?	**JA [ ] NEE [ ]**
C.	Heeft u één of meerdere ouder(e) kind(eren) die ooit eczeem hebben gehad?	**JA [ ] NEE [ ]**

Vraag 7

A.	Heeft u ooit een allergische reactie gehad, bijvoorbeeld galbulten, jeuk of ernstige acute reactie op voedsel of bent u bekend met een voedsel allergie? Zo ja, kunt u aangeven voor welk voedsel?………………………	**JA [ ] NEE [ ]**
B.	Heeft uw echtgenoot / partner ooit een allergische reactie gehad, bijvoorbeeld galbulten, jeuk of een ernstige acute reactie op voedsel of is hij bekend met een voedsel allergie? Zo ja, kunt u aangeven voor welk voedsel?………………………………………………………………………	**JA [ ] NEE [ ]**
C.	Heeft uw één of meerdere ouder(e) kind(eren), die ooit een allergische reactie gehad, bijvoorbeeld galbulten, jeuk of een ernstige acute reactie op voedsel of bekend zijn met een voedsel allergie? Zo ja, kunt u aangeven voor welk voedsel?…………………………………………………………………	**JA [ ] NEE [ ]**

Vraag 8

A.	Heeft u ooit allergisch gereageerd (zoals bovengenoemde reacties) op geneesmiddelen? Zo ja, welke?……………………………………………………	**JA [ ] NEE [ ]**
B.	Heeft uw echtgenoot / partner ooit allergisch gereageerd op geneesmiddelen? Zo ja, welke?………………………………………………………………………	**JA [ ] NEE [ ]**
C.	Heeft u één of meerdere ouder(e) kind(eren) die ooit allergisch hebben gereageerd op geneesmiddelen? Zo ja, welke?………………………………	**JA [ ] NEE [ ]**

Vraag 9

A.	Heeft u ooit allergisch gereageerd (zoals bovengenoemde reacties) op een wespen- of andere insektenbeet?	**JA [ ] NEE [ ]**
B.	Heeft uw echtgenoot / partner ooit allergisch gereageerd op een wespen- of andere insektenbeet?	**JA [ ] NEE [ ]**
C.	Heeft u één of meerdere ouder(e) kind(eren) die ooit allergisch hebben Gereageerd op een wespen- of andere insektenbeet?	**JA [ ] NEE [ ]**

Vraag 10

A.	Heeft u ooit allergische klachten (zoals bovengenoemd (vraag 7) gehad tijdens uitoefening van werk of hobby? Zo ja, kunt u aangeven waarop u allergisch reageerd?…………………………………………………………	**JA [ ] NEE [ ]**
B.	Heeft uw echtgenoot / partner ooit allergische klachten (zoals bovengenoemd (vraag 7) gehad tijdens uitoefening van werk of hobby? Zo ja, kunt u aangeven waarop hij allergisch reageerd?………………………..	**JA [ ] NEE [ ]**
C.	Heeft u één of meerdere ouder(e) kind(eren) die ooit allergisch gereageerd hebben tijdens knutselen, uitoefening van hobby's? Zo ja, kunt u aangeven waarop?………………………………………………………………………	**JA [ ] NEE [ ]**

Dit is het einde van de vragenlijst. Hartelijk dank voor het invullen!

Bijlage II

Informatiebrief m.b.t. de PANDA studie:
"Primaire preventie van astma en allergie door perinatale toedieningen van probiotica"

Geachte Mevrouw, Heer,

U heeft gereageerd op de advertentie waarin u gevraagd wordt deel te nemen aan een wetenschappelijk onderzoek naar de mogelijkheden om de ontwikkeling van astma en allergie reeds op zeer jonge leeftijd te voorkomen. Het is voor deelname aan wetenschappelijk onderzoek vereist dat u een schriftelijke verklaring geeft dat u volledig over het onderzoek bent ingelicht en dat u bereid bent om mee te werken. Dit wordt "informed consent", ofwel "geïnformeerde toestemming genoemd. U zult uitgelegd krijgen wat de opzet is van het onderzoek, wat uw medewerking precies zal inhouden en wat de mogelijke risico's zijn. Ook zal u worden uitgelegd hoe er met de resultaten van het onderzoek wordt omgegaan zodat uw privacy gewaarborgd is. In de nu volgende tekst kunt u nalezen wat deelname aan het onderzoek inhoudt.

Achtergrond van het onderzoek
Astma en allergie komen steeds meer voor in Westerse landen, met name bij kinderen. We denken dat dit komt doordat kinderen en mensen in het algemeen tegenwoordig te weinig afweer ontwikkelen tegen bijvoorbeeld bacteriën, of met andere woorden, dat we tegenwoordig te "schoon" leven. Dat astma en allergie mogelijk iets met het doormaken van infecties te maken heeft, blijkt uit de waarneming dat in ontwikkelingslanden, waar kinderen veel (ernstige) infecties doormaken, er relatief weinig astma en allergie voorkomt. Het is daarom misschien mogelijk om met onschuldige bacteriën (waar je dus niet ziek van wordt) het afweersysteem te beïnvloeden. Dit zou betekenen dat je door toediening van onschuldige bacteriën aan kinderen die een grote kans hebben astma of allergie te krijgen, ervoor kan zorgen dat deze kinderen daar geen of minder last van zullen krijgen.
De onschuldige bacteriën die in dit onderzoek gebruikt gaan worden heten probiotica. Dit zijn bacteriën die normaal ook te vinden zijn (als onschuldige bacteriën) in onze darm.

Doel van het onderzoek
Het uiteindelijke doel van dit onderzoek is om de ontwikkeling van astma en allergie te voorkomen of te verminderen bij kinderen die een hoog risico hebben op het ontwikkelen hiervan. Hierbij zijn we op zoek naar een eenvoudige en veilige manier om deze aandoeningen te voorkomen. Door probiotica of placebo (= geen probiotica) te geven aan zwangere vrouwen in de laatste 8 weken van de zwangerschap en vervolgens aan hun pasgeborenen gedurende 1 jaar en deze 2 groepen (wel of geen probiotica) met elkaar te vergelijken, kunnen we onderzoeken of de kinderen die wel probiotica hebben gekregen inderdaad geen of minder klachten van astma of allergie krijgen dan de kinderen die geen probiotica hebben gehad.

Belang van het onderzoek
In de afgelopen 20-30 jaar komen astma en allergie steeds meer voor in Westerse landen. Voor ons in Nederland betekent dit dat 1 op de 3 kinderen tot de leeftijd van 18 jaar in meer of mindere mate klachten krijgt van astma of allergie (bijvoorbeeld voedselallergie, hooikoorts). Met name kinderen, van wie de moeder klachten heeft van astma of allergie of uit gezinnen waarin de vader plus een ouder kind deze klachten hebben, hebben een verhoogd risico (30-70%) om ook klachten van astma of allergie te krijgen. Tot nu toe zijn we hebben we alleen maar medicijnen die klachten van astma of allergie kunnen verminderen. We kunnen astma of allergie niet genezen. Daarom is het belangrijk om te onderzoeken of we astma en allergie bij kinderen op een veilige en eenvoudige manier kunnen voorkomen en daar is dit onderzoek voor bedoeld.

Wie kunnen er mee doen?
Kinderen met een verhoogde kans om het ontwikkelen van astma of allergie kunnen meedoen aan dit onderzoek. Dit zijn kinderen met een astmatische of allergische moeder, of een astmatische of allergische vader én een astmatisch of allergisch broertje of zusje. De onderzoekster zal voordat u toestemming geeft voor deelname aan dit onderzoek een vragenlijst van astma en allergie met u doornemen of u of uw gezin kunnen deelnemen aan het onderzoek. Omdat we deze kinderen zo vroeg mogelijk bloot willen stellen aan de probiotica en in navelstrengbloed willen kijken naar tekenen van allergie, willen we de moeders al tijdens de zwangerschap probiotica gaan geven. Daarom vragen we (aanstaande) ouders al tijdens de zwangerschap of ze met hun kind mee willen doen.

Methoden en duur van de studie
Voor een goede opzet van wetenschappelijk onderzoek is het nodig om twee groepen te maken, namelijk een groep die probiotica krijgt (probiotica groep) en een groep die geen probiotica krijgt (placebo groep). Het lot bepaalt of u en uw kind in de probiotica groep of in de placebo groep komen. Om het onderzoek zo eerlijk mogelijk te laten verlopen weten zowel u als de onderzoekster niet in welke groep u en uw kind zitten. Het onderzoek duurt in totaal 2 jaar (totdat uw kind 2 jaar is geworden).

Wat gebeurt er voor en tijdens de behandeling
Twee maanden voor de uitgerekende datum wordt een intakegesprek gepland. Daarin wordt het onderzoek uitgebreid besproken. Afhankelijk van de groep waarin u terecht bent gekomen krijgt u de laatste 8 weken van de zwangerschap probiotica of placebo om dagelijks in te nemen. U moet hiermee stoppen na de bevalling. Vanaf het moment dat uw kind geboren is en zijn eerste voeding gaat krijgen moet hij / zij ook dagelijks probiotica of placebo door u toegediend krijgen. Het maakt hierbij niet uit of u borstvoeding of flesvoeding geeft. U bent dus vrij om te kiezen tussen borstvoeding of flesvoeding. Tot de leeftijd van 1 jaar moet uw kind dagelijks de probiotica of placebo van u krijgen. Na zijn / haar eerste verjaardag moet u stoppen met het toedienen hiervan. Tijdens de 2 jaar van het onderzoek wordt 3 maal de polikliniek van het Wilhelmina Kinderziekenhuis van het UMC Utrecht bezocht. Als u kind 3 maanden, 1 jaar en 2 jaar is komt u voor een bezoek naar het ziekenhuis. Er worden dan vragenlijsten afgenomen, er wordt lichamelijk onderzoek gedaan en bloed afgenomen (na verdoving van de huid met zalf). Op de leeftijd van 2 jaar zal er bij u kind met huidpriktesten onderzoek gedaan worden naar allergie. Tevens zullen we u vragen om regelmatig (vanaf dag 2 post partum 1x/week gedurende 4 weken, na staken probiotica/placebo op leeftijd 12 maanden 1x/week gedurende 4 weken, afname 1 faecesportie op de leeftijd van 3, 18 en 24 maanden) wat ontlasting van uw kind in te sturen. Indien u interesse heeft in deelname aan dit onderzoek, zal de onderzoekster met u het schema van de studie uitgebreid doornemen. Wilt u meer informatie dan kunt u haar telefonisch bereiken zoals vermeldt aan het einde van deze informatiebrief.

Wat zijn de mogelijke voor- en nadelen
Deze studie is verbonden met het recent geopende Centrum voor Kinderallergologie.
Aangezien uw kind een verhoogd risico heeft op het ontwikkelen van astma of allergie is het voordeel van deelname aan deze studie dat eventuele klachten hiervan vroegtijdig onderkend kunnen worden, dat er vroegtijdig adequate behandeling gestart kan worden en zo nodig contacten kunnen worden gelegd met kinderarts, dermatoloog of diëtiste.
Er zijn voor u geen nadelen verbonden aan deze studie. Voor uw kind zijn de nadelen dat hij / zij tijdens het onderzoek drie maal het ziekenhuis moet bezoeken voor lichamelijk onderzoek en bloedprikken. De belasting van het lichamelijk onderzoek is minimaal. Om de nadelen van het prikken zo minimaal mogelijk te houden wordt uw kind geprikt door iemand met ervaring in het prikken van kinderen. Tevens zal de plek waar geprikt zal worden eerst verdoofd worden met zalf.

Wat zijn de risico's van deelname aan dit onderzoek
Er zijn geen risico's verbonden voor u of uw kind aan deelname aan dit onderzoek. Probiotica zijn zoals eerder vermeldt, onschuldige bacteriën die normaal in de darmflora voorkomen. Probiotica worden algemeen beschouwd als veilig bij volwassenen en kinderen. Daarnaast worden probiotica steeds meer gebruikt in alledaagse zuivelproducten. De bacteriën die u en uw kind in dit onderzoek zullen krijgen zijn reeds veelvuldig gebruikt in zuivelproducten en hiervan zijn geen infecties of andere vervelende bijwerkingen van bekend.

Vervolgonderzoek
Omdat de diagnose van astma en allergie op de leeftijd van 2 jaar lastig te stellen is, is het mogelijk dat we u in de toekomst verzoeken om deel te nemen aan een kort vervolgonderzoek, waarbij we uw kind éénmalig op de leeftijd van 5 jaar terug zullen zien.

Vertrouwelijkheid van gegevens
Uw persoonsgegevens zullen niet worden gebruikt. U krijgt bij deelname aan dit onderzoek een zogenaamd studienummer, waaronder alle gegevens die verzameld worden tijdens dit onderzoek zullen worden opgeslagen. Alleen de onderzoekers hebben toegang tot de informatie die uw persoonsgegevens verbind aan uw studienummer. Tevens zullen bij eventuele publicatie van onderzoeksgegevens uw persoonsgegevens niet gebruikt worden, waardoor uw privacy gewaarborgd blijft. De gegevens en materialen, zoals bloed, verzameld tijdens dit onderzoek zullen voor geen ander doel gebruikt worden dan dit onderzoek. Overgebleven materialen

zullen wel worden opgeslagen onder vermelding van uitsluitend uw studienummer om eventuele nieuwe vragen tijdens deze studie in te kunnen onderzoeken.

Stoppen van de studie
Uw deelname en die van uw zoon / dochter aan deze studie is geheel vrijwillig en u en uw zoon / dochter kunnen zich op ieder moment uit dit onderzoek terugtrekken. Uw behandelend arts heeft het recht, ook zonder uw toestemming, deze studie of deelname van u en uw zoon / dochter aan de studie op ieder moment te stoppen. Wanneer u besluit niet deel te nemen of wanneer u of uw zoon / dochter zich vroegtijdig terugtrekt uit het onderzoek, zal dit geen gevolgen hebben voor uw of uw zoon / dochter behandeling in het UMC Utrecht.

Aansprakelijkheid
Het UMC Utrecht heeft, als verrichter (opdrachtgever) van dit onderzoek, een risicoverzekering afgesloten voor proefpersonen die meedoen aan wetenschappelijk onderzoek. Voor meer informatie over de verzekering verwijs ik u naar de bijlage behorend bij deze brief.

Klachten
Hoe te handelen bij klachten?
Als u klachten heeft over het onderzoek, kunt u dit melden aan de onderzoeker. Wilt u dit liever niet, dan kunt u contact opnemen met het Bureau Patiëntenservice.
Patiëntenservice locatie WKZ is te vinden op de eerste verdieping naast het opnamebureau, tel. 030-2504468

Administratieve gang van zaken
Dit onderzoek is voorgelegd aan en goedgekeurd door de Raad van Bestuur van het UMC Utrecht op advies van de Medisch Ethische Toetsingscommissie en zal uitgevoerd worden volgens de richtlijnen van de ‘Verklaring van Helsinki’ (Amendement van Edinburgh 2000).

Nadere informatie
Wanneer u nog vragen heeft over dit onderzoek kunt u zich wenden tot de onderzoekers:
Titia Niers, arts-assistent Kindergeneeskunde UMCU, 030-2504000, pieper 5063
Dr. M.O. Hoekstra, kinderarts UMCU, 030-2504001.

Tevens kunt u uitgebreide informatie over dit onderzoek vinden om de website www.pandastudie.nl

Indien u wilt overleggen met een onafhankelijk arts, die niet bij de uitvoering van het onderzoek betrokken is, kunt u contact opnemen met Dr. T. Wolfs, kinderarts UMCU,
030-2504001.

Tenslotte hartelijk dank voor uw aandacht en eventuele medewerking.

Met vriendelijke groet,

Titia Niers, arts-assistent Kindergeneeskunde, UMCU
Dr. M.O. Hoekstra, kinderarts, UMCU

Bijlage III

Toestemmingsverklaring voor deelname aan de PANDA studie:
" Primaire preventie van astma en allergie door perinatale toediening van probiotica"

Er is mij uitgelegd wat het doel en de opzet van deze studie zijn. Ik heb de tijd gehad om een en ander te overdenken en vragen te kunnen stellen over deelname van mezelf en mijn aanstaande zoon / dochter * aan deze studie.

Ik begrijp dat deelname aan dit onderzoek geheel vrijwillig is en dat ik op elk moment mijn toestemming in kan trekken.

Ik weet dat mijn medische gegevens en de medische gegevens over mijn zoon / dochter *, die van betekenis zijn voor dit onderzoek, gebruikt worden voor wetenschappelijke doeleinden en eventueel gepubliceerd worden. Hiermee ga ik akkoord op voorwaarde dat de privacy van mij en van mijn zoon / dochter * gewaarborgd wordt.

Ik wil dat mijn ……………………………………….. wel / niet* op de hoogte wordt gesteld van mijn deelname en de deelname van mijn zoon /dochter * aan deze studie.

Ik wil wel / niet* geïnformeerd worden over de resultaten van het onderzoek na afronding van het onderzoek.

Ik heb uitgelegd gekregen dat mijn zoon / dochter na afloop van dit onderzoek opnieuw benaderd kan worden voor mogelijke vervolgonderzoek op latere leeftijd. Ik geef hierbij mijn toestemming dat dit verzoek gedaan mag worden.

Ik geef hierbij uit vrije wil toestemming dat ik en mijn zoon / dochter * deelnemen aan het onderzoek.

Naam Ouder / Vertegenwoordiger 1.: ………………………………………..
Datum: ………………………………………..
Handtekening: ………………………………………..

Naam Ouder / Vertegenwoordiger 2.: ………………………………………..
Datum: ………………………………………..
Handtekening: ………………………………………..

Ik, ondergetekende, bevestig hierbij dat deze studie zowel mondeling als schriftelijk aan de ouder(s) of wettelijke vertegenwoordiger van bovengenoemde deelnemer is uitgelegd.

Naam Arts / Onderzoeker: ………………………………………..
Datum: ………………………………………..
Handtekening: …………………………………………..

* Doorhalen wat niet van toepassing is.

Bijlage IV

Bijlage bij proefpersooninformatie indien de proefpersonenverzekering van het UMC Utrecht van toepassing is voor het onderzoek (PANDA studie)

Geachte heer, mevrouw,

U denkt er over na om mee te doen aan een wetenschappelijk onderzoek waaraan in meer of mindere mate risico's verbonden zijn. In verband met de Wet Medisch wetenschappelijk Onderzoek met mensen treft u hierbij aanvullende informatie aan betreffende de verzekering die in verband met de studie ten behoeve van alle proefpersonen is afgesloten.

Het UMC Utrecht heeft, als verrichter (opdrachtgever) van dit onderzoek[1)], een risicoverzekering afgesloten voor proefpersonen die meedoen aan wetenschappelijk onderzoek. De verzekering is afgesloten bij Marketform Limited te Londen onder het polisnummer L030082.

Deze verzekering dekt schade door dood of letsel die het gevolg is van deelname aan het onderzoek, en die zich gedurende de deelname aan het onderzoek openbaart, of binnen vier jaar na beëindiging van de deelname aan het onderzoek. De schade wordt geacht zich te hebben geopenbaard wanneer deze bij de verzekeraar is gemeld.

De verzekeraar van het onderzoek is:
Naam: Marketform Ltd
Adres: One Lime Street 783, LONDON EC3M 7HA

In geval van schade kunt u contact op nemen met de schaderegelaar:
Naam: C en E Verzekeringen
De heer H. Melinga
Adres: Herculesplein 5
Telefoonnummer: 030 - 25 60 406
Fax: 030 - 25 60 250
E-mail: verzekering@cene.nl

De verzekering biedt een maximum dekking van € 450.000 per proefpersoon en € 3.500.000 voor het gehele onderzoek, en € 5.000.000 per jaar voor alle onderzoeken van dezelfde opdrachtgever. De dekking van specifieke schades en kosten is verder tot bepaalde bedragen beperkt. Dit is opgenomen in het Besluit verplichte verzekering bij medisch-wetenschappelijk onderzoek met mensen. Informatie hierover kunt u vinden op de website van de Centrale Commissie Mensgebonden Onderzoek: www.ccmo.nl.

Voor deze verzekering geldt een aantal uitsluitingen. De verzekering dekt niet:
- schade waarvan op grond van de aard van het onderzoek zeker of nagenoeg zeker was dat deze zich zou voordoen;
- schade aan de gezondheid die ook zou zijn ontstaan indien u niet aan het onderzoek had deelgenomen;
- schade die het gevolg is van het niet of niet volledig nakomen van aanwijzingen of instructies;

[1)] Doorhalen indien niet van toepassing.

- schade aan nakomelingen, als gevolg van een nadelige inwerking van het onderzoek op u of uw nakomeling;
- bij onderzoek naar bestaande behandelmethoden: schade die het gevolg is van één van deze behandelmethoden;
- bij onderzoek naar de behandeling van specifieke gezondheidsproblemen: schade die het gevolg is van het niet verbeteren of van het verslechteren van deze gezondheidsproblemen.

Bijlage V

SERIOUS ADVERSE EVENTS (SAE) REPORT

Instructies: Vul dit formulier volledig in z.s.m. na melding van ongewenste bijwerking of SAE en stuur het formulier ter beoordeling op naar Dr. T. Wolfs en METC UMC Utrecht (zie pag. 2)

Melding: ☐ Initieel ☐ Follow-up<br>Datum:…/………/……..<br>Plaats:…………………<br>Studienummer deelnemer:……………………<br>Initialen deelnemer:……………………………<br>Geboortedatum:………/……………/…………..<br>Geslacht: ☐ man ☐ vrouw<br><br>Formulier ingevuld door:………………………	Persoonsgegevens onderzoeker:<br>Naam:………………………………………………………<br>Adres:……………………………………………………<br>……………………………………………………………..<br>Telefoon:…………………………………………………<br>E-mail:……………………………………………………<br>Handtekening onderzoeker:<br><br>Datum:………../……………../………………………

Beschrijving van adverse event:

Datum **start symptomen:** …../…../…… Datum **einde bijwerking:**…../…../…..

**Ernst**	*Relatie tot studie product **	*Studie status:* ***status van deelnemer tgv bijwerking/SAE***	**Uitkomst**
☐ Mild<br>☐ Matig<br>☐ Ernstig<br>☐ Levensbedreigend	☐ Definitief<br>☐ Waarschijnlijk<br>☐ Mogelijk<br>☐ Waarschijnlijk Niet<br>☐ Geen relatie	☐ Toediening product reeds afgerond<br>☐ Toediening product voortgezet<br>☐ Toediening product gestaakt<br>☐ Deelname studie gestopt door onderzoeker	☐ Duurt nog voort<br>☐ Rest verschijnselen<br>☐ Hersteld<br>☐ Overlijden<br>☐ Onbekend

***** Als toevoeging, denkt u dat de bijwerking/SAE gerelateerd is aan één van het volgende. Zo ja, licht dit toe :

- ❑ Ander product gelijktijdig ingenomen met studieproduct Ja ☐ Nee ☐
- ❑ Procedure van toediening Ja ☐ Nee ☐
- ❑ Andere verdachte oorzaak Ja ☐ Nee ☐

Kruis alles aan wat betreft de bijwerking/SAE van toepassing is:

- ❑ Resulteerde in overlijden…../…../….. (datum van overlijden)
- ❑ Was levensbedreigend
- ❑ Leidde tot ziekenhuisopname
- ❑ Leidde tot blijvende of tijdelijke handicap
- ❑ Was medisch niet relevant

SERIOUS ADVERSE EVENTS (SAE) REPORT (vervolg)

Datum…../…../……	Studienummer deelnemer……………

**Studie product:** Geef details over het studie product, of controle als de interventie geblindeerd is				
Studie product	Hoeveelheid	Route	Schema	Datum
			Eerste gift<br>Tweede gift<br>Derde gift<br>Andere gift	…../…../…..<br>…../…../…..<br>…../…../…..<br>…../…../…..
**Toegevoegd product:** Geef details over het product dat ten behoeve van toediening van het studie product wordt toegevoegd aan het studie product.				
Toegevoegd product	Hoeveelheid	Route	Schema	Datum
			Eerste gift<br>Tweede gift<br>Derde gift<br>Andere gift	…../…../…..<br>…../…../…..<br>…../…../…..<br>…../…../…..
Ruimte voor aanvullende opmerkingen				

Stuur het volledig ingevulde formulier naar Dr. T. Wolfs, Algemene Kindergeneeskunde, huispostnummer KE 04 133.1 en een copy van het formulier naar de METC UMC Utrecht, huispostnummer D 01.343.
