# Supplementary material for: Long Term Development of Gut Microbiota Composition in Atopic Children: Impact of Probiotics
Source: PLoS One. 2015 Sep 17;10(9):e0137681. doi: 10.1371/journal.pone.0137681 (PMC4574972; doi:10.1371/journal.pone.0137681)
Supplement: S2 File — (PDF) [file pone.0137681.s003.pdf]

PANDA STUDY

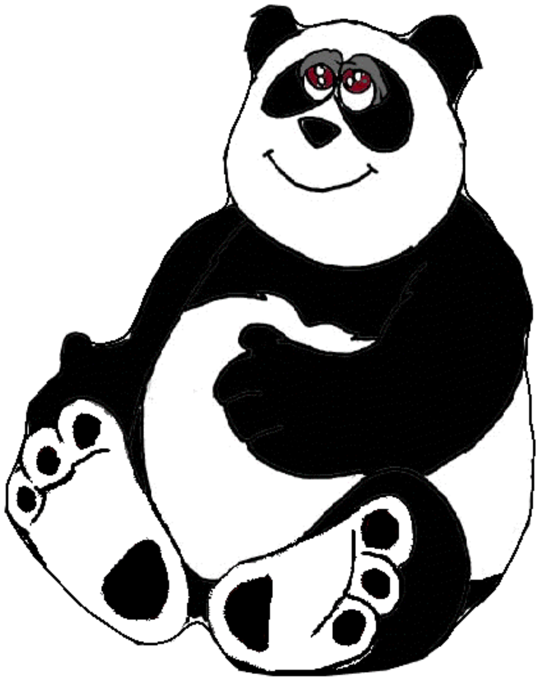

Probiotics AND Allergy

Primary prevention of asthma and allergy
by supplementation of probiotics in early life

ENGLISH SUMMARY

The date of the original protocol was January 2004

SUMMARY

Background. Atopic diseases are increasing in countries with a Western lifestyle. The hygiene hypothesis states that the increase in atopic disease could be due to reduced exposure to microbial antigens in early in life. In search of new preventive therapies for atopic disease, exposure of pregnant women with previous or recent atopic disease, and their offspring to probiotics has been suggested. Probiotics are mono or mixed cultures of microbes which, when applied to animal or man, can beneficially affect the host, among others by inducing an immune response. Probiotics are generally accepted to be safe in children. Probiotics have shown to be effective in primary prevention of atopic disease in high-risk neonates in one study so far. However, it is still unclear by what mechanism probiotics work and which is the most immunopotent (combinations of) probiotic(s). It is likely that antigen-presenting cells (APC's) are involved, since these cells are important in the first line of defence in the gastrointestinal tract. It can be imagined that the immune response is the result of the interplay between probiotics and APC's. In particular, the match between pathogen-associated molecular patterns (PAMP's) on probiotics and their counterparts on APC's, the pathogen-recognition-receptors (PRR's) (like for instance Toll-like receptors) is decisive in this aspect.

Hypothesis. Administration of probiotics to pregnant women and their offspring may reduce the development of sensitization as well as the onset of atopic disease in their offspring.

Aim. To study the effect of probiotics on sensitisation and the prevalence of atopic disease, the severity of atopic disease, the intestinal flora and immune parameters in high-risk newborns.

Methods. To study this hypothesis, a randomised, double-blind placebo-controlled trial will be carried out by administration of probiotics to pregnant women with previous or recent atopic disease as well as to their offspring. Primary outcome parameters are firstly the prevalence and severity of sensitization and atopic disease in the offspring during a follow-up of two years. Secondary outcome parameters are the change in stool composition during treatment with probiotics and in-vitro production of cytokines by PBMCs collected at 3 months, 1 year and 2 years of age.

Expected results. Perinatal administration of probiotics to pregnant women and their offspring may hamper the development of sensitization and atopic disease in their offspring. This may be due to modulation of the intestinal microbiota composition, and modulation of the developing immune system.

DESCRIPTION OF THE STUDY:

Background

Allergy

Atopic disease adds considerably to childhood morbidity. The cumulative prevalence during childhood is estimated to be 20 to 30%.[1] Moreover, in several countries (especially with a Western lifestyle) the prevalence of atopic disease has increased during the last decades.[2] This increase in atopic disease may be explained by a decreased exposure to microbial antigens, which are presumed to be obligatory for a ''normal'' development of the innate and adaptive immune system (*the hygiene hypothesis*).[3] Allergy is considered to be an immune-mediated disease: the inflammatory response is characterised by a Th2-predominance.[4] Increased exposure to microbial antigens by administration of probiotics may be a way to down-regulate the Th2-response. As a result, the increasing prevalence of atopic and immune-mediated disease may be suppressed as well.[3]

Probiotics

Probiotics are defined as ''live microbial, food ingredients that are beneficial to health by promoting the endogenous host defense mechanisms''.[5] Probiotics are generally considered to be safe since they belong to the normal human intestinal flora.[6] In children with acute infectious diarrhoea, probiotics are generally accepted to be effective in reducing both quantity and frequency of the diarrhoea. Moreover, in these trials probiotics have proved to be safe; no adverse effects have been reported.[7] Circumstantial evidence for a beneficial effect of probiotics on atopic disease comes from epidemiological studies. Björksten has shown that the prevalence of atopic disease was lower in children from Estland compared to Sweden, and that the faeces of the former group contained more Lactobacilli, which is a probiotic.[8] Subsequently, in a randomised controlled trial Kalliomaki showed that administration of Lactobacillus GG to pregnant women during the last 6 weeks of gestation and subsequently in their high-risk offspring during the first six months of life reduces the incidence of atopic eczema by almost 50%.[9]Recently, four year follow-up data were published. At the age of four the odds ratio for eczema was still around 50% for the intervention group compared to the placebo group.[10] No difference in the incidence of asthma and rhinitis and number of positive skin prick tests was found between the two groups. Suggesting that treatment with LAB GG results in a reduction of the rate of non-atopic eczema rather than atopic dermatitis.[11] Nevertheless, the exact mechanism by which probiotics exert their effect is as of yet unknown. Several mechanisms have been proposed to contribute to the probiotic effect, mostly generated from animal studies: direct interfering with antigen transport by stabilisation of the gut microflora, enhancement of the humoral immune response (thereby promoting the intestine's immunologic barrier) and stimulation of the non-specific host resistance to microbial pathogens (thereby stimulating immune elimination).[12,13]

Antigen-Presenting-Cells (APC's)

APC's are among the first line of defence in the mucosa of the human body. In the inflammatory response, APC's mediate between the environment and the second line of defence, the orchestrating T-cells. Recently, it has been suggested that 'pattern recognition receptors' on APC's can recognise microbes carrying specific pathogen-associated-molecular-patterns (PAMP's). One example of such a 'pattern-recognition-receptor (PRR)' is the family of Toll-like receptors (TLR's), which may play an important role in the contact between APC's and probiotics.[14,15] Polymorphisms in TLR's have indeed been described in humans.[16] Therefore, it can be imagined that polymorhisms in TLR's contribute, and may even be decisive, to the occurrence of disease, as well as to the individual response to treatment. It has long been believed that only T-cells orchestrate the direction of the immune response.[17] Recently, this view has been adapted, since APC's can be divided into type1- and type2-cells. By differentiation into either APC1 (Dendritic cell type 1 – DC1-cell) or APC2 (DC2- cell), these cells can skew the immune response into a Th1- or Th2-direction.[13] Moreover, DC's can induce regulatory T-cells. Therefore, it is current belief that APC's not only transfer signals from antigens to T-cells, but that they actively contribute to the direction of the inflammatory response.[18] Since APC's from the lungs and the gut cannot be obtained non-invasively, APC's will be developed in vitro from monocytes from cord blood, as previously described.[19] We assume that pre-APC's from atopic patients are already primed due to the presence of cytokines and chemokines in utero.

T-cells

T-cells can be divided into suppressor T-cells and helper T-cells (Th-cells). Th-cells are characterised as Th1-cells and Th2-cells based on their production of cytokines. Th1-cells mainly produce IL2 and IFNγ and are involved in intracellular infections. Th2-cells mainly produce IL4, IL5 and IL-13 and are mediators in extracellular infections, helminth infections and atopic disease. Within this Th1/Th2-concept two principles are important: Th1- and Th2-cells stimulate their own proliferation and stimulation (autocrine stimulation) and inhibit each others proliferation and differentiation (reciproque inhibition).[20] In atopic disease, the immune response is skewed towards a Th2-response.[15] Although the Th1/Th2-concept is useful in understanding the pathophysiology and considering therapeutic strategies, it is in general too simplified to explain the pathophysiology of inflammatory diseases. Recently, the presence of Th0-, Th3- and Tr1-cells has been shown.[21] Th0 cells are considered to be immature T-cells, not yet differentiated with respect to cytokine production. Th3- and Tr1-cells are regulatory T-cells (T-reg cells), formerly called suppressor T-cells, which play an important role in the development of tolerance. In that way, these T-reg cells contribute to immune homeostasis. Th3-cells can be induced by pulmonary DC's that produce IL10, and Tr1-cells can be induced by DC's from the gut by production of TGFβ. Th3- and Tr1-cells may downregulate the immune response by production of cytokines.[22]

The ontogeny of allergic disease

The intrauterine environment is Th2-skewed in all pregnancies to prevent the foetus from stillbirth. The development of sensitisation is, therefore, not thought to be due to Th2-skewing, but to an inability to respond to environmental stimuli that must evoke the necessary switch to Th1/Th2 equilibrium.[23] One of the cells necessary to realise this switch may be the regulatory T-cell.[24,25] It can be hypothesised that sensitisation takes place due to insufficient formation of regulatory T-cells. Therefore, acquisition of an increased number of regulatory T-cells could be essential in preventing or suppressing the onset of allergic disease in high-risk new-borns.

Primary or Tertiary (treatment) prevention of allergic disease

So far, it has been accepted that allergy and asthma are inflammatory diseases dominated by a Th2-immune response. Prevention or treatment of these diseases should consist of suppressing the Th2-response or the induction of a counteractive Th1-response. Several IFNγ-generating (i.e. Th1-inducing) treatment strategies have been described, like administration of live or dead bacteria, CpG-oligodinucleotides or combinations of interleukines, either alone or in combination with allergen (allergen-specific immunotherapy). Besides IFNγ-generation, induction of Th2-cell anergy or Th2-cell suppression by induction of regulatory T-cells are suggested.[26,27] If allergic disease is caused by a failure of early instigation of regulatory T-cells, than induction of these cells could be the aim of primary prevention of allergic disease. Probiotics have shown to be associated with a decline in the prevalence of atopic dermatitis in infancy. This could be due to the induction of regulatory T-cells in the intestine, which secrete chemo- and cytokines that downregulate Th2-responses on APC- and T-cell–level. The hypothesis of induction of regulatory T-cells by probiotics is substantiated by increased levels of TGF-β (produced by regulatory T-cells) in breast milk of pregnant women receiving probiotics.[28]

Timing

Several studies indicate that sensitisation may already take place in utero.[29,30] It has so far not yet been decided whether pre- or postnatal administration of probiotics is equally effective. To modulate successfully the immune system of new-borns, there seems to be a "window of opportunity" within the first six months of life. However, to modulate the immune system by influencing the colonisation of the intestinal flora the first week of life is crucial[.31]

Aim

The aim of this study is firstly to establish the effect of early administration of probiotics on the development and severity of allergic disease in the offspring. Secondly, we will study the effect on the microbial colonisation of the intestinal tract, the effect on the T-cell response and the association with the TLR-repertoire on APC's .

Hypotheses

Administration of probiotics to allergic pregnant women and their high risk-offspring results in:

- a reduction of sensitisation and incidence or severity of atopic disease in the offspring

  through:

- a permanent presence of probiotics in the intestine, followed by induction of regulatory T cells in the intestinal wall, the draining lymph nodes and in the peripheral blood, depending on the presence of the TLR-repertoire on APC's.

Research questions
Does administration of probiotics to pregnant women with allergic disease and to their high-risk offspring result in:
1. a reduction of the prevalence and severity of allergic disease?
2. a permanent presence of probiotics in the intestine?
3. a T-reg cell response in the peripheral blood of the infants?
4. Does the development of this response depend on the presence and function of a certain repertoire of TLR's on APC's?

Design, methods and timetable
Study Design
This study concerns a randomised, double-blind, placebo-controlled clinical trial in pregnant women with previous or recent allergic disease and their offspring at risk for the development of allergic disease (see below). Probiotics or placebo will be administered to the pregnant women on a daily basis from four before the expected delivery. Newborns will receive either probiotics or placebo, depending to which group the mother has been at random allocated, during their first year of life. Follow-up is done untill the second birthday.

Study Design – Time Table

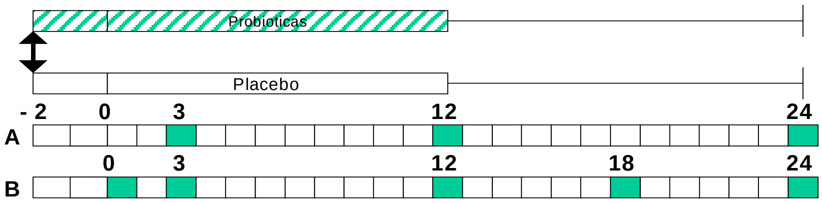

- Time indicated in months
- 0 = time of birth
- Randomisation at time point –2 months
- A = follow-up visits + peripheral blood investigations
- B = collecting fecal samples

Patients
Inclusion criteria
Eligible are pregnant women who ever or recently suffered from allergic disease, like food allergy, allergic eczema, asthma or rhinitis. Or families in which the father as well as at least one sibling suffers from allergic disease.
Families in which an older child is affected with cow's milk allergy can not be included because in the Netherlands it is generally accepted to start from birth with hydrolysed formulas in siblings.

Exclusion criteria
Antenatal systemic use of immunomodulatory drugs, like corticosteroids.
Regular use of products containing probiotics.

Feeding
Participating women are allowed to choose between breast-feeding or infant formula. In case of infant formula, participants will be asked to agree on using the same formula among all non-breast-feeding participants, in order to avoid to much heterogeneity in feeding.

Methods
Independent variables
Probiotics (strain(s) will be selected based on the results of the animal study [8] and the in-vitro study [9]. Probiotics will be administered to mothers from 4 weeks before expected delivery and to the neonates till the end of the first year of life, in a daily dose of $1*10^{10}$ cfu. Probiotics will be prepared and provided by Winclove Bioindustries bv, Amsterdam.

Execution
Once inclusion is secured an appointment is made at the 7th month of pregnancy. At that time, pregnant women will visit the out-patient department (OPD), inclusion is formalised and women are allocated to one of the study groups by computer randomisation. Participants start using probiotics and fill in daily record cards at home to monitor the use of any diet and drugs. By telephone, the father will be asked to inform the researcher or one of the senior investigators about the birth of the offspring. Just after birth, cord blood will be collected (by the obstetric nurse, the general practitioner) and will be brought to the laboratory to determine base-line immune parameters for the offspring (we have ample experience with this procedure in the BCG-study). The newborn will be allocated to the same treatment group (either probiotics or placebo) as the mother. The father can collect the study drug for the newborn. The parents fill in a daily record card for the baby with respect to symptoms of infectious and allergic disease as well as the use of any diet and drugs. When the child reaches the age of 3, 12 months and 2 years the parents visit the OPD for a medical history, physical examination and blood sampling. The general practitioner will be asked by mail to deliver information on diagnosis of any allergic disease.

Outcome variables
All variables will be determined in the offspring. The outcome variables will be described in order of the research questions

1. Does administration of probiotics to pregnant women with allergic disease and to their high-risk offspring result in a reduction of the prevalence and severity of allergic disease in the offspring ?

The incidence of allergic disease is the primary outcome criteria and is defined as follows (*Laan MP, et al. Markers for early sensitisation and inflammation in relation to clinical manifestations of atopic disease up to 2 years of age in 133 high-risk children. Clin Exp Allergy 2000;30:944-53*) :

- **eczema** – eczema (or atopic dermatitis) is based on Sampson's criteria: erythema, oedema, oozing and excoriation with evidence of itchiness lasting longer than 4 weeks (*Sampson HA. Pathogenesis of eczema. Clin Exp Allergy 1990;20:459-67*). The severity will be measured by using the Scoring Atopic Dermatitis (SCORAD) (*Sprikkelman AB, et al. Severity scoring of allergic dermatitis: a comparison of three scoring systems. Allergy 1997;52:944-9*).
- **food allergy** – food allergy is diagnosed clinically by the presence of erythematous, papular, macrovesicular skin eruptions, diarrhoea, vomiting, or respiratory problems in relation to food ingestion. For the definite diagnosis an elimination/provocation/re-elimination procedure is necessary. A positive skin test or increased specific IgE are considered supportive.
- **asthma** – asthma is difficult to define in children younger than 2 years.. For bronchusobstructive disease in infants younger than 3 years of age, 3 different phenotypes can be distinguished. (*Martinez FD. Development of wheezing disorders and asthma in pre-school children. Paediatrics 2002;109(S2):362-7*). Of these, recurrent bronchus-obstructive complaints in the presence of atopy (defined as increased specific serum IgE) is likely to proceed to chronic asthma. Therefore, atopy present at or before the age of 2 in combination with recurrent periods of bronchusobstruction will be designated as asthma. At the age of 2, general practitioners as well as parents will be telephoned to make a definite diagnosis of asthma in each child prior to breaking the study randomisation code. A change in asthma will be scored as a decrease in the use of short acting β_2 agonists. Since a more definite diagnosis of asthma can be made at the age of 6, a follow-up of this cohort of children will be done. However, this will not be consistent with the duration of this project. Therefore, at the age of 6 diagnosis of asthma and other allergic disease will be evaluated by a ISAAC-questionnaire sent to the parents by mail.
- **rhinitis** – rhinitis is defined as recurrent periods of watery rhinorrhoea in combination with frequent sneezing and nasal itching, redness and tearing of the eyes. The diagnosis is confirmed by the

presence of either a positive skin test or an increased specific serum IgE. A change in disease severity will be scored as a decreased need of relieving drugs.

Symptom score
A daily record card will be kept, containing data on allergic disease, infectious disease (including gastro-enteritis), doctor’s visits (and diagnosis), drug prescriptions and use, change of feeding habits.
A detailed medical history will be taken with focus on symptoms of allergic disease on every planned visit to the out-patient department. A change in severity of allergic disease will be scored by a change in the use of drugs.

Physical examination
This will be performed every planned visit to the out-patient department with focus on asthma and allergic disease. A change of eczema will be evaluated using the SCORAD.

2. Does administration of probiotics to pregnant women with allergic disease and to their high-risk offspring result in a permanent presence of probiotics in the intestine ?
The presence of probiotics in the intestine will be measured as the microbial composition of the infants stool.

In order to answer this question we will collect:
a. post-partum cultures of nose, anus, throat and umbilicus swabs.
b. from the second day post partum faecal sample once weekly, for 4 weeks
c. at the age of 12 months after ending the administration of probiotics again faecal samples once weekly for 4 weeks
d. at the age of 3, 18 and 24 months faecal sample once

The human intestinal microflora is dominated by anaerobic micro-organisms, that are laborious and time-consuming to culture, and consequently many are still not yet cultured or described. Thus, the present study will use rapid modern molecular methods, based on the 16S rRNA gene, that are culture independent to monitor changes in the intestinal flora during the trial.

PCR and denaturing gradient gel electrophoresis (DGGE) of 16S ribosomal DNA genes.
(Favier CF, Vaughan EE et al. Molecular Monitoring of Succession of Bacterial Communities in human neonates. Appl Environ Microb 2002;68:219-26)
This is a qualitative analysis by which dominant species in faecal samples can be determined and monitored during time. Faecal samples are collected by the parents at home, send to the hospital and frozen until further use. DNA will be isolated from faecal samples. Bacterial 16S rRNA will be amplified with PCR and the PCR fragments are separated by DGGE. The resulting profiles are used to monitor changes in the total microbiota over time.

Within this technique, specific bacterial populations like *Bifidobacteria* and *Lactic acid bacteria* can be determined with species specific PCR and DGGE. This implicates that the administered probiotic strain can be monitored.

Fluorescent *in situ* hybridisation (FISH) combinated with flow cytometric analysis.
(Vaughan EE et al. The intestinal Labs Antonie van Leeuwenhoek 2002;82;341-52)
This is a quantitative analysis to identify and enumerate bacteria in complex ecosystems. Bacteria in the faeces are hybridised with fluorescent oligonucleotides (16S rRNA) probes, specific for a group of bacteria. Currently, six validated probes are available and among those for *Bifidobacteria, Lactic acid bacteria, Clostridrium and Bacteroides*. With flow cytometric analysis labelled bacterial species can be differentiated from unlabeled bacterial populations.

3. Does administration of probiotics to pregnant women with allergic disease and to their high-risk offspring result in a T-reg cell response in the peripheral blood of the infants ?
Baseline parameters for participating neonates will be determined in cord blood. Follow-up visits are planned at the age of 3, 12 and 24 months, and peripheral blood will be drawn at these visits. Parameters will be described below.

IgE
Total and specific IgE concentrations will be determined by immunoCAP technique (Pharmacia, The Netherlands). Specific IgE against the 6 major allergens (milk, house dust mite, cat, dog, grass- and tree pollen) will be determined.

Cytokine determination
Cytokines will be determined in unstimulated and stimulated peripheral blood cells. Fresh blood will be diluted with RPMI medium (GIBCO/BRL). Cells will be stimulated with LPS and IFN-γ. After 18 hours, supernatant is collected for determination of cytokines. Cells will also be stimulated with medium (RPMI), CD2/CD28 and PHA. After 48 and 72 hours, supernatant will be collected. Proliferation of the cells will be determined at 48 and 72 hours with incorporation of 3H-thymide. The following cytokines will be determined by *Luminex* (de Jager et al., submitted): monoclonal antibody (specific for a certain cytokine) is bound specifically to Luminex polystyren microsheres (with a specific fluorescence pattern). Binding of the corresponding cytokine is visualized with a biotine labled second antibody, directed against a non-overlapping epitope on the same cytokine. After incubation with streptavidine-phycoerythrine the fluorescence-intensity of the spheres is measured on the Bioplex system (BioRad). Because different Luminex spheres display different fluorescence characteristics, they can be used in a mixture (multiplex). In one sample (volume 50 microliter) 15 different cytokines can be determined simultaneously: interleukin 1α (IL-1α), IL-1β, IL-2, IL-4, IL-5, IL-6, IL-8, IL-10, IL-12p70, IL-13, IL-15, IL-17, IL-18, γ-interferon and TNF-α. The sensitivity of the assay is comparable with a conventional ELISA, intra-assay CV <10%, interassay CV < 20%.

Morphology of mononuclear cells
To determine the phenotype of lymphocytes, mononuclear cells will be double stained with CD3, CD4, CD8, CD45RA, CD45RO. To differentiate between Th1 en Th2-cells, expression of CD30, CCR3, CCR4, CCR5 and CCR8 will be determined. *Regulatory T-cells* will be differentiated using the markers CD4, CD25, CTLA-4, GITR, L-selectin, CCR-4, CD45RB and CD45RO. In regulatory T-cells, all these markers are expressed.

4. Does the development of this response depend on the presence and function of a certain repertoire of TLR's on APC's ?
APC's will be generated from the cord blood derived mononuclear cells from each participant, as we did in the research carried out this year in the in-vitro project (''*A comparison of the in-vitro effects of several strains of probiotics on antigen-presenting cells derived from cord-blood mononuclear cells derived from atopic and non-atopic children"*). In short, cord blood-derived monocytes will be seeded in 24 well culture plates (Costar, Cambridge, MA) at a density of $0.5*10^6$ cells/ml. After 1 h incubation at 37C, non-adherent cells will be removed and adherent cells will be cultured in IMDM (Life Technologies, Paisley, UK) and 1% FCS (Hyclone, Logan, UT) supplemented with GM-CSF (500 IU/ml) and IL-4 (250 U/ml) to obtain DC. After 3 days the media including the supplements will be refreshed. After 6 days of culture, DC will be harvested and washed extensively before use. Purity and quality of the DC will be determined by FACS analysis using mAb against CD1a, CD3, CD14, CD40, CD115 and CD83. APC's will be frozen and stored untill usage.

Subsequently the presence (expression) of TLR's on APC's will be determined by flow cytometry using antibodies to TLR's (currently commercially available: TLR2, -3, -4, -6 and -9).

The function of TLR's on APC's will be determined in-vitro by combining each participant's APC's, T-cells and exposing these cells to the probiotic used in this study. The T-cell response in the supernatants will be determined by Luminex as described above.

Statistics
The primary outcome parameter is the incidence of atopic disease in the high-risk offspring. For the power analysis we anticipate a 25% reduction in the cumulative prevalence of eczema in the intervention group, which implicates a number of 50 children in each of the treatment groups. This results in the inclusion of 120 pregnant high-risk women, including 20% drop-out. The SPSS package will be used to process the data. Stratification of the data will be done for maternal smoking, for current maternal eczema, and for infant feeding.

Probiotic Product

Probiotics are livemicro-organisms which, when administered in adequate amounts, confer a health benefit on the host (WHO 2002). Their precise mode of action is not well established, but they have been shown to reduce markers of intestinal inflammation and intestinal permeability in disease states, and this might change the way in which antigens present in the intestine are recognised by the immune system.

Selection criteria

In collaboration with Winclove Bio Industries B.V., Amsterdam, a multispecies probiotic product is designed based on the individual strain properties, mainly stability, surviving the GI-tract and antibiotic resistance. Out of 75 strains, 14 strains were selected (see Preliminary Research) for further characterization. A multispecies product was designed (in stead of a single strain) because of the possible symbiotic, strengthening effect the strains have on each other.

Selected strains

1. Bifidobacterium bifidum
2. Bifidobacterium Infantis
3. Lactococcus lactis

Administration

The probiotic product will be supplied once daily in a total amount of 3 x 10^9 Colony Forming Units (CFU) (which means 1 x 10^9 CFUs of each strain). The supplement will be dispensed as a stable powder in identical individually packed sachets containing 3 g of material. The contents of each sachet can be mixed with at least 10 ml of water, breast milk or infants formula, depending on the parents' preference, and ingested as a suspension.

The placebo product consists of the carrier of the probiotic product, i.e. rice starch and maltodextran.

Safety

Probiotics are generally accepted to be safe in children, and a number of probiotic strains are already incorporated in baby formulas. The frequently applied probiotic strains (Bifidobacteria, Lactobacilli) are considered to be commensal microorganisms, without pathogenic properties. Only well-known bacterial strains will be administered, used in food industry for producing food supplements and dairy products (cheese, yoghurt, quark). In general, probiotics are well accepted without major side effects. Feeling unwell because of excessive production of gas can occur. In case of Adverse Events during the treatment period, the Ethics Committee will directly be informed. Supplementation of the product can of course be terminated when necessary.

Preliminary Research

"In-vitro effects of probiotics on proliferation, morphology and cytokine production on cord-blood derived mononuclear cells from high-risk newborns".

In the *cord blood in-vitro study* we aimed to answer the question which probiotic(s) are the most immunopotent on human cells. Probiotics were provided by Winclove Bio Industries bv, Amsterdam. Firstly, a selection of probiotics (14 strains) was based on tolerance to acid and bile, and adherence to the intestinal epithelium and inhibition of pathogens. Secondly, we investigated the effect of these strains by determination of cytokine production and proliferation of PBMCs from healthy volunteers. Results show that the different strains can be characterised by different profiles of cytokine production by PBMC's. Based on the assumption that the cytokine environment in both the intestinal tract and dendritic cells determines the direction of the immune response, we have further selected 3 strains with either a high production of IL-10 leading to a regulatory T-cell response, or strains that lead to a high production of pro-inflammatory cytokines or strains that induce a Th1 cell response.

Finally, we will culture APC's from cord-blood derived mononuclear cells from offspring from atopic and non-atopic mothers. These APC's will be exposed to the 3 different strains of probiotics and their influence on the cord-blood derived T-cells with respect to cytokine production will be determined. Based on these results we will choose which (combination) of probiotics strain(s) will be used in the primary prevention study.

Ethics

This study will be performed in accordance with the ethical standards laid down in the 1964 Declaration of Helsinki and its later amendments and after approval by the Medical Ethics Committee of the UMC Utrecht. The UMC enclosed a subject insurance for scientific research participants.
Parental written informed consent will be obtained.

Effectuation

The study will be conducted by Ms. L.E.M. Niers, Pediatric Resident and PhD candidate. The research project will be supervised by Dr. M.O. Hoekstra and Dr. G.T. Rijkers.
All persons concerned in the project are listed in the adjective table (see Dutch protocol).

REFERENCES (from the original Dutch protocol)

Hygiene hypothesis

1. Umetsu DT, McIntire JJ, Akbari O et al. Asthma: an epidemic of dysregulated immunity. Nat Immunol 2002;3:715-20.
2. Von Mutius E. Pro: the increase in asthma can be ascribed to cleanliness. Am J Respir Crit Care Med 2001:164:1106-7.
3. Platts-Mills TA, Woodfolk JA, Sporik RB. Con: the increase in asthma cannot be ascribed to cleanliness. Am J Respir Crit Care Med 2001;164:1107-8.
4. Weiss ST. Eat dirt: the hygiene hypothesis and allergic disease. N Eng J Med 2002;347:930-1.
5. Wills-Karp M, Santeliz J, Karp CL. The germless theory of allergic disease: revisiting the hygiene hypothesis. Nat Rev Immunol 2001;1:69-75.

Timing: sensitization in utero and early life

6. Bjorksten B. Allergy priming early in life. Lancet. 1999;353:167-8.
7. Warner JO, Jones CA, Kilburn SA et al. Prenatal sensitization in humans. Pediatr Allergy Immunol 2001;11S13:6-8.
8. Herz U, Joachim R, Ahrens B et al. Allergic sensitization and allergen exposure during pregnancy favor the development of atopy in the neonate. Int Arch Allergy Clin Immunol 2001;124:193-6.

Allergic disease in childhood

9. Laan MP et al. Markers for early sensitization and inflammation in relation to clinical manifestations of atopic disease up to 2 years of age in 133 high-risk children. Clin Exp Allergy 2000;30:944-53.
10. Sampson HA. Pathogenesis of eczema. Clin Exp Allergy 1990;20:459-67.
11. Sprikkelman AB et al. Severity scoring in allergic dermatitis:a comparison of three scoring systems. Allergy 1997;52:44-9.
12. Martinez FD. Development of wheezing disorders and asthma in preschool children. Pediatrics 2002;109(S2):362-7.

Role of the intestinal flora in the development of allergy and atopy

13. Bjorksten B, Naaber P, Seppe E et al. The intestinal microflora in allergic Estonian and Swedish 2-year-old children. Clin Exp Allergy. 1999;29:342-6.
14. Kalliomaki M, Kirjavainen P, Eerola E et al. Distinct patterns of neonatal gut microflora in infants in whom atopy was and was not developing. J Allergy Clin Immunol. 2001;107:129-34.
15. Kalliomaki M, Isolauri E. Role of intestinal flora in the development of allergy. Curr Opin Allergy Clin Immunol 2003;36:223-7.

Reviews or editorials addressing the use of probiotics

16. Mursh SH. Toll of allergy reduced by probiotics. Lancet 2001;357:1057-9.
17. Isolauri E, Sutas Y, Kankaanpaa P et al. Probiotics: effects on immunity. Am J Clin Nutr. 2001;73:44S-50S
18. Erickson KL, Hubbard NE. Probiotic immunomodulation in health and disease. J Nutr. 2000;130:403S-9S
19. Matricardi PM. Probiotics against allergy: data, doubts and perspective. Allergy 2002;57:185-7.

In-vitro effects of probiotics

20. Hessle C, Hanson LA, Wold AE. Lactobacilli from human gastrointestinal mucosa are strong stimulators of IL-12 production. Clin Exp Immunol. 1999;116:276-82.
21. Christensen HR, Frokiaer H, Pestka JJ. Lactobacilli differentially modulate expression of cytokines and maturation surface markers in murine dendritic cells. J Immunol. 2002;168:171-8.
22. Miettinen M, Vuopio-Varkila J, et al. Production of human tumor necrosis factor alpha, interleukin-6 (IL-6), and IL-10 is induced by lactic acid bacteria. Infect Immun.1996;64:5403-5.
23. Miettinen M, Matikainen S, Vuopio-Varkila J et al. Lactobacilli and streptococci induce interleukin-12 (IL-12), IL-18 and gamma interferon production in human peripheral blood mononuclear cells. Infect Immun 1998;66:6058-62.
24. Pessi T, Sutas Y, Hurme M et al. Interleukin-10 generation in atopic children following oral Lactobacillus Rhamnosus GG. Clin Exp Allergy 2000;30:1804-8.
25. Pochard P, Gosset P, Grangette C et al. Lactic acid bacteria inhibit Th2 cytokine production by mononuclear cells from allergic patients. J Allergy Clin Immunol 2002;110:617-23.

Clinical trials probiotics in allergic disease

26. Kalliomaki M, Salminen S, Poussa T et al. Probiotics and prevention of atopic disease: 4 year follow-up of a randomised placebo-controlled trial. Lancet 2003;361:1869-71. (*primary prevention*)
27. Kalliomaki M, Salminen S, Arvilommi H et al. Probiotics in primary prevention of atopic disease: a randomised placebo-controlled trial. Lancet 2001;357:1076-79. (*primary prevention*)
28. Kirjavainen PV, Salminen SJ, Isolauri E. Probiotic bacteria in the management of atopic disease: underscoring the importance of viability. J Pediatr Gastroenterol Nutr 2003;36:223-7.
29. Majamaa H, Isolauri E. Probiotics: a novel approach in the management of food allergy. J Allergy Clin Immunol. 1997;99:179-85.

30. Isolauri E, Arvola T, Sutas Y et al. Probiotics in the management of atopic eczema. Clin Exp Allergy. 2000;30:1604-10.
31. Kalliomaki M, Ouwehand A, Arvilommi H et al. Transforming growth factor-beta in breast milk: a potential regulator of atopic disease at an early age. J Allergy Clin Immunol. 1999:104:1251-7.
32. Rautava S, Kalliomaki M, Isolauri E. Probiotics during pregnancy and breastfeeding might confer immunomodulatory protection against atopic disease in the infant. J Allergy Clin Immunol 2002;109:119-21.
33. Rosenfeldt V, Benfeldt E, Nielsen SD et al. Effect of probiotic Lactobacillus strains in children with atopic dermatitis. J Allergy Clin Immunol 2003;111:389-95.
34. Helin T, Haahtela S, Haahtela T. No effect of oral treatment with an intestinal bacterial strain, Lactobacillus rhamnosus (ATCC 53103), on birch-pollen allergy: a placebo-controlled double-blind study. Allergy. 2002;57:243-6.

Gene by environment interaction

35. Smit JJ, Loveren H van, Hoekstra MO et al. Influence of the macrophage bacterial resistance gene, Nramp1 (Slc11a1), on the induction of allergic asthma in the mouse.
FASEB J 2003 17:958-60.

Safety of probiotics

36. Borriello SP, Hammes WP, Holzapfel W et al. Safety of probiotics that contain lactobacilli and bifidobacteria. Clin Infect Dis. 2003;36:775-80.
37. Sipsas NV, Zonios DI, Kordossis T. Safety of Lactobacillus strains used as probiotic agents. Clin Infect Dis 2002; 34: 1283-1284
