# Supplementary material for: Long Term Development of Gut Microbiota Composition in Atopic Children: Impact of Probiotics
Source: PLoS One. 2015 Sep 17;10(9):e0137681. doi: 10.1371/journal.pone.0137681 (PMC4574972; doi:10.1371/journal.pone.0137681)
Supplement: S3 File — (PDF) [file pone.0137681.s004.pdf]

RESEARCH PROTOCOL

‘Evaluation of the effect of probiotics on development of asthma and allergic rhinitis during childhood – follow-up study at age 5 or 6 years’

(August 2009)

PROTOCOL TITLE ‘*<Evaluation of the effect of probiotics on development of asthma and allergic rhinitis during childhood – follow-up study at age 5 or 6 years.l>*’

**Protocol ID**	**NL**
**Short title**	**Follow-up study on probiotics and atopy**
**Version**	**1.0**
**Date**	**August 2009**
**Coordinating investigator/project leader**	**Dr. C.K. van der Ent**<br>**088-7553201**<br>**K.vanderEnt@umcutrecht.nl**
**Principal investigator(s) (in Dutch: hoofdonderzoeker/uitvoerder)**<br>***Multicenter research: per site***	**Dr. C.K. van der Ent**<br>**088-7553201**<br>**K.vanderEnt@umcutrecht.nl**
**Sponsor (in Dutch: verrichter/opdrachtgever)**	**Dr. C.K. van der Ent**<br>**088-7553201**<br>**K.vanderEnt@umcutrecht.nl**
**Independent physician(s)**	**Dr. T. Wolfs**<br>**088-7554555**<br>**T.Wolfs@umcutrecht.nl**
**Laboratory sites <*if applicable*>**	**Laboratory of Paediatric Immunology, Wilhelmina Kinderziekenhuis, Utrecht**
**Pharmacy <*if applicable*>**	**Not applicable**

PROTOCOL SIGNATURE SHEET

**Name**	**Signature**	**Date**
**For non-commercial research,**<br>**Head of Department:**<br>**Prof. dr. W. Kuis**		
**Coordinating Investigator/Project leader/Principal Investigator:**<br>**Dr. C.K. van der Ent**		

TABLE OF CONTENTS

1. INTRODUCTION AND RATIONALE 8
2. OBJECTIVES 10
3. STUDY DESIGN 11
4. STUDY POPULATION 12
4.1 Population (base) 12
4.2 Inclusion criteria 12
4.3 Exclusion criteria 12
4.4 Sample size calculation 12
5. TREATMENT OF SUBJECTS 13

6. INVESTIGATIONAL MEDICINAL PRODUCT 14
7. METHODS 15
7.1 Study parameters/endpoints 15
7.1.1 Main study parameter/endpoint 15
7.1.2 Secondary study parameters/endpoints (if applicable) 15
7.1.3 Other study parameters (if applicable) 15
7.2 Randomisation, blinding and treatment allocation 15
7.3 Study procedures 15
7.4 Withdrawal of individual subjects 16
7.4.1 Specific criteria for withdrawal (if applicable) 16
7.5 Replacement of individual subjects after withdrawal 16
7.6 Follow-up of subjects withdrawn from treatment 16
7.7 Premature termination of the study 16
8. SAFETY REPORTING 17
8.1 Section 10 WMO event 17
8.2 Adverse and serious adverse events 17
8.2.1 Suspected unexpected serious adverse reactions (SUSAR) 18
8.2.2 Annual safety report 18
8.3 Follow-up of adverse events 18
8.4 Data Safety Monitoring Board (DSMB) 18
9. STATISTICAL ANALYSIS 19
9.1 Descriptive statistics 19
10. ETHICAL CONSIDERATIONS 20
10.1 Regulation statement 20
10.2 Recruitment and consent 20
10.3 Objection by minors or incapacitated subjects (if applicable) 20
10.4 Benefits and risks assessment, group relatedness 20
10.5 Compensation for injury 21
10.6 Incentives (if applicable) 21
11. ADMINISTRATIVE ASPECTS AND PUBLICATION 22
11.1 Handling and storage of data and documents 22

11.2 Amendments....................22
11.3 Annual progress report....................22
11.4 End of study report....................22
11.5 Public disclosure and publication policy....................23
12. REFERENCES....................24

LIST OF ABBREVIATIONS AND RELEVANT DEFINITIONS

ABR	ABR form (General Assessment and Registration form) is the application form that is required for submission to the accredited Ethics Committee (ABR = Algemene Beoordeling en Registratie)
AE	Adverse Event
AR	Adverse Reaction
CA	Competent Authority
CCMO	Central Committee on Research Involving Human Subjects
CV	Curriculum Vitae
DSMB	Data Safety Monitoring Board
EU	European Union
EudraCT	European drug regulatory affairs Clinical Trials GCP Good Clinical Practice
IB	Investigator’s Brochure
IC	Informed Consent
IMP	Investigational Medicinal Product
IMPD	Investigational Medicinal Product Dossier
METC	Medical research ethics committee (MREC); in Dutch: medisch ethische toetsing commissie (METC)
(S)AE	Serious Adverse Event
SPC	Summary of Product Characteristics (in Dutch: officiële productinfomatie IB1-tekst)
Sponsor	The sponsor is the party that commissions the organisation or performance of the research, for example a pharmaceutical company, academic hospital, scientific organisation or investigator. A party that provides funding for a study but does not commission it is not regarded as the sponsor, but referred to as a subsidising party.
SUSAR	Suspected Unexpected Serious Adverse Reaction
Wbp	Personal Data Protection Act (in Dutch: Wet Bescherming Persoonsgevens)
WMO	Medical Research Involving Human Subjects Act (Wet Medisch-wetenschappelijk Onderzoek met Mensen

SUMMARY

Rationale: Probiotics, if instituted early in life, can give a significant reduction of development of eczema in infants at high risk of atopic diseases. In 2004 a randomized controlled trial started to investigate whether probiotics have a significant effect on the development of atopic symptoms as eczema, allergic rhinitis, food allergy and asthma. This study population has been followed until the age of two years. The present study is meant as a prolongation of the former study, we will perform a follow-up study of the included children on development of atopic diseases, in particular asthma and allergic rhinitis.

Objective: To establish the effect of perinatal probiotics on the prevalence of allergic disease at the age of 5 years.

Study design: Prospective, single blind, randomised, placebo-controlled trial.

Study population: Subjects are 98 predominantly Caucasian high risk children, having either a mother, or both a father and at least one sibling with past or present atopic diseases, to whom probiotics or placebo was administered perinatally during the previously carried out PANDA study.

Intervention (if applicable): None.

Main study parameters/endpoints: Development of allergic symptoms, evaluated by questionnaire and physical examination. Development of allergic astma and allergic rhinitis, measured by lung function testing, nitric oxide measurements in exhaled air, and specific IgE to allergens.

Nature and extent of the burden and risks associated with participation, benefit and group relatedness: Parents and child are asked to fill in a questionnaire, and pay one visit to the out patient department for physical examination, blood sampling and pulmonary function testing with measurement of exhaled nitric oxide. The risks of these procedures are negligible and the burden minimal. It should be carried out specifically in these children, since they have been included in the former study and received placebo or probiotics.

INTRODUCTION AND RATIONALE

The last decennia a rising incidence of allergy and atopic disorders is seen, especially amongst children. The aim of this study is to investigate possible primary prevention in high risk children by means of adminstration of probiotics from 4-6 weeks prenatally until the age of one year.

The hypothesis is, that early administration of probiotics to pregnant women with allergy and subsequently to their offspring, leads to prevention or reduction of development of sensitisation and allergic disorders in childhood.

Modification of the intestinal flora early in life by administration of probiotic bacteria may be a potential approach to prevent atopic disease. Strachan (1) suggested a decreased occurrence of T helper type 1 (Th1)-associated infections to be a causative factor in the increased prevalence of allergy. More recently, modifications of the hygiene hypothesis have moved away from the impact of Th1-driving infections and towards the influence of the diversity of the microbial exposure in general during establishment of the intestinal flora (2,3). In industrial countries having a high degree of hygiene, changed microbial exposure might cause an altered composition of the intestinal flora and thus, an altering of the immunomodulatory microbial signalling posed during the critical stages of immune development in infancy (4). Atopic disease is the result of a T helper type 2 (Th2)-deviated immune respons characterized by the production of the cytokines IL-4, IL-5, and IL-13. According to the hygiene hypothesis, insufficient infection-related Th1 responses during the neonatal period and infancy were considered to lead to unrestricted Th2 responses, resulting in allergic diseases.
Because the gastro-intestinal (GI) tract encompasses the largest surface of the human body where microbial products interact with the immune system, much attention is focused on the possibility that the intestinal flora may play a role in the development of atopic disease (2). Indeed, different bacterial colonization patterns have been observed in the intestinal flora of allergic children when compared with non-allergic children. Lactobacilli and bifidobacteria are more commonly found in the intestinal flora of non-allergic children, while allergic children seem to harbour higher number of clostridia and staphylococci (5,6). Lactobacilli and bifidobacteria are most frequently investigated probiotic species for possible beneficial effects in prevention and treatment of allergic disease (7). Preliminary data indicate that probiotic bacteria can be effective in prevention and treatment of atopic disease (8-13). Perinatal administration of probiotic bacteria (*Lactobacillus rhamnosus GG*) significantly reduced the development of eczema during the first 2-4 years of life in children with a family history of atopic disease (8,9). In addition, specific probiotic strains have been beneficial in the treatment of infants suffering from atopic eczema and cow’s milk allergy by significantly decreasing the SCORAD score (10,11,14). The mechanism by which probiotic bacteria may modify immune-mediated diseases such as atopic disease, and which different species or strains may contribute are poorly understood.

Niers et al(15,16) showed the ability of live lactic acid bacteria to modulate the immune responses by inducing IL-10 in peripheral blood mononuclear cells, and these effects appeared to be strain specific. Based on this study, they selected *B. Bifidum*, *B. infantis*, and *Lc Lactis* (because of their good IL-10-inducing capacity as well as efficient inhibition of IL-5 and IL-13) to be used as a multi-species probiotics in a clinical trial on primary prevention of allergic diseases by probiotic bacteria. *B. Bifidum* was most potent to polarize dendritic cells to drive Th1 cell responses involving increased IFN-gamma producing T cells concomitant with reduction of IL-4-producing T cells. *L. Lactis* was much less effective in this respect.

This study will encompass a follow-up of the previously included children in the above mentioned double blinded randomized clinical trial performed by Niers from 2003 till 2007. It will be carried out when the children have reached the age of 5 years. Since Niers completed follow-up till the age of 2 years, a thorough evaluation of the development of asthma and allergic rhinitis was not possible. The diagnosis of asthma is best made at an age beyond 5 years for reasons of lung function testing, and the prevalence of allergic rhinitis rises from the age of 4. The aim of this follow-up study is to study if administration of probiotics during pregnancy and in the first year of life has any influence on the prevalence of asthma and allergic rhinitis, in Caucasian children at high risk of developing atopy, at the age of 5 or 6 years.

Besides this, we wish to perform a pilot study evaluating the diagnostic value of measurement of nasal nitric oxide (nNO) in the diagnosis of allergic rhinitis. An effect of allergic rhinitis on nNO is not consistently reported. However, most studies suggest that nNO is increased in allergic rhinitis [12-17]. Normal values for nNO in children are well defined by Struben et al [18]. De Winter et al will shortly publish an evaluation of methodology for nNO-measurements, with good reproducibility and applicability, investigated at the Department of Paediatric Pulmonology of our hospital [19]. We do not expect a direct relationship between probiotics and nNO, but want to support the diagnosis of allergic rhinitis. This test is easily available in our hospital, easy to perform and not experienced as invasive by the child. As we expect a direct relationship between the severity of allergic rhinitis and level of nNO, we wish to evaluate the predictive value of the measurement of nNO for the diagnosis of allergic rhinitis.

1. OBJECTIVES

Primary Objective:
The aim of the present study is to determine the effect of probiotics in high-risk infants on the prevalence and severity of asthma and allergic rhinitis, lung function and specific IgE at the age of 5 or 6 years.

2. STUDY DESIGN

A randomised double-blind placebo-controlled prospective study was carried out in 156 high-risk children. 98 children completed the intervention period and the two years of follow-up. The present study comprises a follow-up at the age of 5 or 6 years, in the outpatient clinic.

3. STUDY POPULATION

3.1 Population (base): Children who previously participated in the PANDA study

3.2 Inclusion criteria: Children who previously participated in the PANDA study

3.3 Exclusion criteria: None

3.4 **Sample size calculation:** We will try to get as little as possible loss to follow-up by trying to find all the former participants of the PANDA-study and to ask for the collaboration.

For the pilot study evaluating the diagnostic value of measurement of nasal nitric oxide, we will evaluate the complete cohort, not considering whether the child has received probiotics. We estimate the prevalence of allergic rhinitis to be 15% in this cohort. To be able to detect a statistical significant difference between nasal NO in the subjects with a diagnosis of allergic rhinitis from the questionnaire and subjects who do not, we will need a minimum of 10-15 participants with allergic rhinitis, and the same amount who do not suffer from allergic rhinitis. In evaluating this, we consider the means for NO in both groups to be 450 and 600 ppb, the standard deviation 115 ppb, the power 90% and the alfa 0.05. With the expected prevalence of allergic rhinitis (14 of 95 participants), this should be possible.

4. TREATMENT OF SUBJECTS

Not applicable

5. INVESTIGATIONAL MEDICINAL PRODUCT

Not applicable

6. METHODS

6.1 Study parameters/endpoints

6.1.1 Main study parameter/endpoint

The prevalence of allergic disease, focussing on the prevalence of asthma and allergic rhinitis.
Definition of asthma: Periodic wheezing and dyspnoea, combined with a 9% reversibility in FEV1 and/or exhaled NO above 20 ppb.
Definition of allergic rhinitis: Periodic or perennial sneezing, runny, or blocked nose when not having a cold or the flu.

6.1.2 Secondary study parameters/endpoints (if applicable)

Specific IgE to allergens.

6.1.3 Other study parameters (if applicable)

Not applicable.

6.2 Randomisation, blinding and treatment allocation

The previous study was double-blinded, this was abated at the end of the PANDA study. In this follow-up study, the researcher will be blinded, but parents are not.

6.3 Study procedures

The questionnaire is based on the ISAAC questionnaire. It will be used to evaluate the participants for symptoms indicative of asthma, allergic rhinitis, eczema and food allergy at the age of 5 or 6 years, with the most used and best comparable questionnaire of nowadays research in allergy.
The treating physician will be asked for doctor-diagnosed atopic diseases (asthma, allergic rhinitis, food allergy, eczema, urticaria).
Physical examination.
Baseline pulmonary function (forced expiratory volume in 1 second (FEV1), forced vital capacity (FVC), peak expiratory flow (PEF), and subdivisions of forced expiratory flow) will be measured. Airway reversibility will be measured after administration of 400 microgram of salbutamol. Exhaled nitric oxide will be measured, including nasal nitric oxide (nNO). nNO will be measured during breath holding (nNO-BH) and during humming (nNO-HE). nNO-measurement is not experienced as invasive by the child. nNO-BH is measured during breath hold after inhalation to TLC. The subjects will be asked to hold their breath for 10 seconds while air is sampled from the right nostril.

To measure nNO-HE, subjects will be asked to make a humming noise during nasal exhalation at the frequency of a tuning fork. Before nNO-HE measurements subjects are asked not to speak during 5 minutes. After inhalation of ambient air to TLC subjects exhale to FRC through their left nostril, while air is sampled from the right.

The nasal humming procedure will be done at two different frequencies with the assistance of tuning forks of respectively 128 and 440 Hz (nNO-HE_{128} and nNO-HE_{440}, respectively).
Participants will be asked to stop the possible use of nasal corticosteroids for 6 weeks before nNO-measurement, long acting β-mimetica for 48 hours before lung function testing, and short acting β-mimetica for 12 hours before lung function testing, if clinically acceptable for the medical situation of the child. This will be evaluated by the investigator and deliberated with the treating physician.
Specific IgE will be determined by Phadiatop assay, concerning the most common allergens, in 1 ml. of blood.

6.4 Withdrawal of individual subjects

Subjects can leave the study at any time for any reason if they wish to do so without any consequences. The investigator can decide to withdraw a subject from the study for urgent medical reasons.

6.4.1 **Specific criteria for withdrawal (if applicable)** Not applicable.

6.5 **Replacement of individual subjects after withdrawal** Not applicable.

6.6 **Follow-up of subjects withdrawn from treatment** Not applicable.

6.7 Premature termination of the study

No reason to terminate this study prematurely.

7. SAFETY REPORTING

7.1 Section 10 WMO event

In accordance to section 10, subsection 1, of the WMO, the investigator will inform the subjects and the reviewing accredited METC if anything occurs, on the basis of which it appears that the disadvantages of participation may be significantly greater than was foreseen in the research proposal. The study will be suspended pending further review by the accredited METC, except insofar as suspension would jeopardise the subjects' health. The investigator will take care that all subjects are kept informed.

7.2 Adverse and serious adverse events

No serious adverse effects are to be expected, children will undergo physical examination, capillary blood sampling and lung function testing. Mild adverse effects could be dyspnoea or wheezing during the 2 days before the lung function testing, for which the investigator will provide good accessibility for deliberations of the parents. Another mild adverse effect could be exacerbation of allergic rhinitis since intranasal corticosteroids will be stopped during 6 weeks before testing. For this, the investigator can be contacted. Restarting the medication is possible at any time by the child, parents or treating physician and discontinuation of the medication can be rejected at any time by the child, parents or treating physician.

Adverse events are defined as any undesirable experience occurring to a subject during a clinical trial, whether or not considered related to the investigational drug. All adverse events reported spontaneously by the subject or observed by the investigator or his staff will be recorded.

A serious adverse event is any untoward medical occurrence or effect that at any dose results in death;

- is life threatening (at the time of the event);
- requires hospitalisation or prolongation of existing inpatients' hospitalisation;
- results in persistent or significant disability or incapacity;
- is a congenital anomaly or birth defect;
- is a new event of the trial likely to affect the safety of the subjects, such as an unexpected outcome of an adverse reaction, lack of efficacy of an IMP used for the treatment of a life threatening disease, major safety finding from a newly completed animal study, etc.

All SAEs will be reported to the accredited METC that approved the protocol, according to the requirements of that METC.

7.2.1 Suspected unexpected serious adverse reactions (SUSAR)

Not applicable.

7.3 Follow-up of adverse events

Adverse events are not expected to occur during this study.
All adverse events will be followed until they have abated, or until a stable situation has been reached. Depending on the event, follow up may require additional tests or medical procedures as indicated, and/or referral to the general physician or a medical specialist.

7.4 Data Safety Monitoring Board (DSMB)

Not applicable.

8. STATISTICAL ANALYSIS

8.1 **Descriptive statistics:**

For the main endpoint we will calculate the relative risks with 95% confidence intervals.
For the secondary endpoints we will apply parametric and non-parametric tests, depending on the distribution of the parameter.
For the pilotstudy evaluating the diagnostic value of nNO, we will calculate sensitivity, specificity, positive and negative predictive values for nNO in the diagnosis of allergic rhinitis.
All analysis will be performed with the SPSS/PC+ package.

9. ETHICAL CONSIDERATIONS

9.1 Regulation statement

The study will be conducted according to the principles of the Declaration of Helsinki (version 2004) and in accordance with the Medical Research Involving HumanSubjects Act (WMO).

9.2 Recruitment and consent

Subjects will be recruited by drs. D.M.W. Gorissen among the participants of the previous PANDA-study. Parents will be asked if their child is willing to participate. They will receive printed information and will be contacted by phone after one week by the investigator. If they decide to participate, they are asked to return the informed consent form attached to the printed information.

9.3 Objection by minors or incapacitated subjects (if applicable)

Subjects can abandon the research any time. Medical staff, the investigator and the parents will observe the child during the examination, blood sampling and lung function testing to make sure the child is not being forced to co-operate. The Dutch Association of Pediatrics has edited a Behavioural Code concerning Resistance of minors in case of medical scientific research with children (11). This will be applied.

9.4 Benefits and risks assessment, group relatedness

No direct benefit can be expected for the participants, except for a thorough screening for the presence of atopic diseases with if indicated an appropriate reference to a specialist. The risk of blood sampling is negligible, and the same applies to pulmonary function testing including exhaled nitric oxide measurement. For the need of stopping the inhaled beta-mimetics, a risk of dyspnoea and wheezing exists, which will be treated with re-administration of beta-mimetics. The same applies to discontinuation of nasal corticosteroids, which carries the risk of exacerbation of allergic rhinitis. If the condition of the child does not allow stopping the above mentioned medication or the parents are not willing to do so, they can still fullfill the questionnaires but they cannot perform lungfunction testing in case of beta-mimetics, or nasal NO measurement in case of nasal corticosteroids.

We are convinced that the yield of the study for addressing the effect of probiotics on the development of atopic disease exceeds the burden of one visit to the hospital with the above mentioned diagnostic procedures and the risks.

This follow-up study can only be carried out on these subjects, since they have participated in the primary intervention (the PANDA-study) with either probiotics or placebo.

9.5 Compensation for injury

We will insure for the risks of adverse effects of the study to the participants, by use of the general insurance of the University Medical Center of Utrecht.

9.6 Incentives (if applicable)

Subjects will be able to obtain a travelling allowance for the visit to the outpatient department..

10. ADMINISTRATIVE ASPECTS AND PUBLICATION

10.1 Handling and storage of data and documents

The handling of personal data complies with the Dutch Personal Data Protection Act. They will be handled confidentially and anonymously, provided with a code, kept by the investigators drs. D.M.W. Gorissen and dr. C.K. van der Ent.
The sera will be kept for the timespan of the study, which is estimated on 2 years. Thereafter they will be destroyed.

10.2 Amendments

Amendments are changes made to the research after a favourable opinion by the accredited METC has been given. All amendments will be notified to the METC that gave a favourable opinion.

All substantial amendments will be notified to the METC and to the competent authority.

Non-substantial amendments will not be notified to the accredited METC and the competent authority, but will be recorded and filed by the sponsor.
< *Examples of non-substantial amendments are typing errors and administrative changes like changes in names, telephone numbers and other contact details of involved persons mentioned in the submitted study documentation*.>

10.3 Annual progress report

The investigator will submit a summary of the progress of the trial to the accredited METC once a year. Information will be provided on the date of inclusion of the first subject, numbers of subjects included and numbers of subjects that have completed the trial, serious adverse events/ serious adverse reactions, other problems, and amendments.

10.4 End of study report

The investigator will notify the accredited METC of the end of the study within a period of 8 weeks. The end of the study is defined as the last patient's last visit.

In case the study is ended prematurely, the investigator will notify the accredited METC, including the reasons for the premature termination.

Within one year after the end of the study, the investigator/sponsor will submit a final

study report with the results of the study, including any publications/abstracts of the study, to the accredited METC.

10.5 Public disclosure and publication policy

The previous trail has been registrated at ClinicalTrials.gov.

ISRCTN Register: ClinicalTrials.gov Identifier NCT00200954

REFERENCES

1. Strachan DP. Hay fever, hygiene, and household size. BMJ 1989;299:1259-60.
2. Kalliomaki M, Isolauri E. Role of intestinal flora in the development of allergy. Curr Opin Allerg Clin Immunol 2003;3:15-20.
3. Yazdanbakhsh M, Kremsner PG, Ree R. Allergy, parasites, and the hygiene hypothesis. Science 2002;296:490-4.
4. Willis-Karp M, Santeliz J, Karp CL. The germless theory of allergic disease: revisiting the hygiene hypothesis. Nat Rev Immunol 2001;1:69-75.
5. Bjorksten B, Naaber P, Sepp E, Mikelsaar M. The intestinal microflora in allergic Estonian and Swedish 2-year-old children. Clin Exp Allergy 1999;29:342-6.
6. Kalliomaki M, Kirjavainen P, Eerola E, Kero P, Salminen S, Isolauri E. Distinct patterns of neonatal gut microflora in infants in whom atopy was and was not developing. J Allergy Clin Immunol 2001;107:129-34.
7. Ogden NS, Bielory L. Probiotics, a complementary approach in the treatment and prevention of pediatric atopic disease. Curr Opin Allergy Clin Immunol 2005;5:179-84.
8. Kalliomaki M, Salminen S, Poussa T, Arvilommi H, Isolauri E. Probiotics and prevention of atopic disease: 4-year follow-up of a randomized placebo-controlled trial. Lancet 2003;361:1869-71.
9. Kalliomaki M, Salminen S, Arvilommi H, Kero P, Koskinen P, Isolauri E. Probiotics in primary prevention of atopic disease: a randomized placebo-controlled trial. Lancet 2001;357:1076-9.
10. Isolauri E, Arvola T, Sutas Y, Moilanen E, Salminen S. Probiotics in the management of atopic eczema. Clin Exp Allergy 2000;30:1604-10.
11. Majamaa H, Isolauri E. Probiotics: a novel approach in the management of food allergy. J Allergy Clin Immunol 1997;99:179-85.
12. Pessi T, Sutas Y, Hurme M, Isolauri E. Interleukin-10 generation in atopic children following oral Lactobacillus rhamnosus GG. Clin Exp Allergy 2000;30:1804-8.
13. Rautava S, Kalliomaki M, Isolauri E, Probiotics during pregnancy and breast-feeding might confer with immunomodulatory protection against atopic disease in the infant. J Allergy Clin Immunol 2002;109:119-21.
14. Rosenfeldt V, Benfeldt E, Nielsen SD et al. Effect of probiotic Lactobacillus strains in children with atopic dermatitis. J Allergy Clin Immunol 2003;111:389-95.
15. Niers LEM, Timmerman HM, Rijkers GT, Bleek GM van, Uden NOP van, Knol EF, Kapsenberg ML, Kimpen JLL, Hoekstra MO. Identification of strong interleukin-10 inducing lactic acid bacteria which down-regulate T helper type 2 cytokines. Clin Exp Allergy 2005;35:1481-9.

16. Niers LEM, Hoekstra MO, Timmerman HM, Uden NO van, Graaf PMA de, Smits HH, Kimpen JLL, Rijkers GT. Selection of probiotic bacteria for prevention of allergic diseases: immunomodulation of neonatal dendritic cells. Clin Exp Immunol 2007;149:344-52.
